# Impact of Structural Flexibility on Tunneling Contribution to the Reaction Mechanism of Spontaneous Succinimide Formation in Asn-Gly-Containing Peptides

**DOI:** 10.3390/biom16091266

**Published:** 2026-09-01

**Authors:** Fruzsina Pilhál, Bianka Szalainé Ágoston, Imre Jákli, Ernő Keszei, András Perczel

**Affiliations:** 1 Laboratory of Structural Chemistry and Biology, Institute of Chemistry, ELTE Eötvös Loránd University, Pázmány Péter Sétány 1/A, 1117 Budapest, Hungary; 2 Hevesy György PhD School of Chemistry, ELTE Eötvös Loránd University, Pázmány Péter Sétány 1/A, 1117 Budapest, Hungary; 3 HUN-REN–ELTE Protein Modelling Research Group, Institute of Chemistry, ELTE Eötvös Loránd University, Pázmány Péter Sétány 1/A, 1117 Budapest, Hungary; 4 Department of Physical Chemistry and Chemical Kinetics Laboratory, Institute of Chemistry, ELTE Eötvös Loránd University, Pázmány Péter Sétány 1/A, 1117 Budapest, Hungary

**Keywords:** reaction mechanism, deamidation, kinetic isotope effect, tunneling, QM computation, NMR spectroscopy, NG isomerization/isomerisation, structural flexibility, activation energy

## Abstract

Spontaneous deamidation and isomerization of asparagine (Asn) residues is a major pathway of nonenzymatic protein aging, where Asn-Gly-containing peptides (NG motifs) represent the most reactive sequence. In this context, the first step of isomerization is succinimide formation, which is initiated by a concerted proton-transfer and nucleophilic activation step. Although the overall mechanism is established, the detailed nature of the rate-determining succinimide formation step and the possible role of nuclear quantum effects remain unclear. Here, we combine density functional theory (DFT), intrinsic reaction coordinate (IRC) analysis, and quantitative NMR kinetics to investigate hydrogen/deuterium (H/D) substitution effects on NG isomerization in different model peptides, which represent both conformationally flexible and restricted systems. Deuteration preserves the reaction pathway and structural evolution, indicating an invariant classical reaction coordinate. However, kinetic isotope effects in the flexible peptide system reveal a mixed classical–tunneling mechanism in the rate-determining step with observable deviations from classical over-the-barrier behavior. In contrast, the conformationally restricted peptide shows suppressed tunneling contributions. These findings demonstrate that conformational accessibility modulates proton tunneling in spontaneous peptide rearrangements and extends nuclear quantum effects beyond enzymatic systems to nonenzymatic processes associated with protein aging.

## 1. Introduction

Spontaneous deamidation and isomerization of asparagine (Asn) residues represents one of the most widespread nonenzymatic post-translational modifications in proteins, playing a central role in long-term protein aging and structural degradation [1,2,3,4,5,6]. This transformation is particularly relevant in long-lived proteins such as crystallins, hemoglobin, and other slow-turnover structural proteins, where cumulative chemical damage contributes to functional decline over timescales of months to years [7,8,9,10,11]. In addition, NG-mediated (asparagine-glycine, Asn-Gly) deamidation has been implicated in pathological processes associated with protein instability, and has been explored in the context of prodrug activation and chemotherapeutic strategies [12].

**Figure 1:**
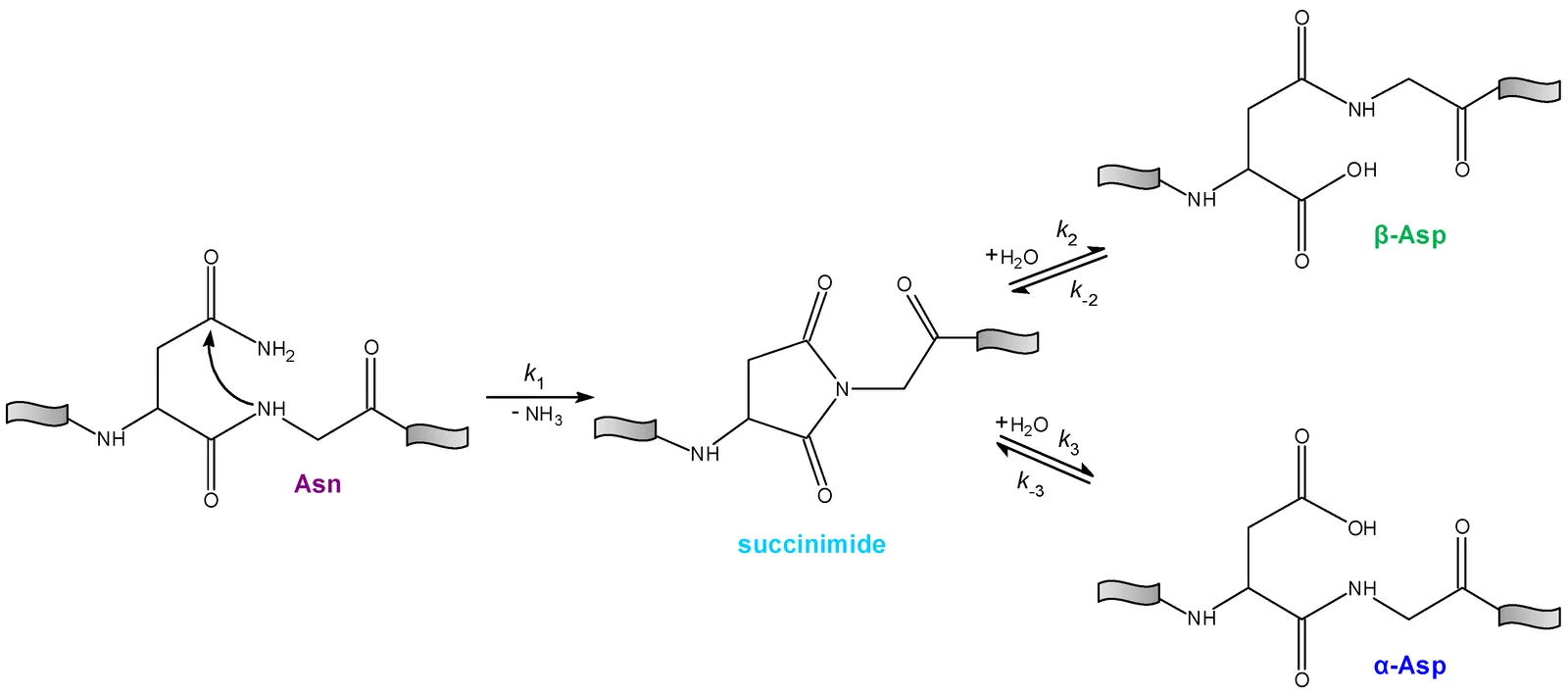
Schematic pathway of NG sequence deamidation, isomerization and rearrangement. The reaction proceeds via intramolecular nucleophilic attack of the Gly backbone amide nitrogen on the Asn side chain carbonyl carbon, followed by cyclization to a five-membered succinimide intermediate, deamidation and subsequent hydrolysis to yield both β- and α-aspartic acid products. The β-Asp isomer corresponds to a backbone-elongated isoaspartic acid residue containing an additional methylene insertion within the peptide backbone, whereas the α-Asp form retains the canonical backbone connectivity. The succinimide-forming cyclization represents the rate-determining and isotope-sensitive step of the overall isomerization pathway, making it suitable for mechanistic investigation.

At the molecular level, NG motifs constitute the most reactive sequence context for deamidation [13]. The reaction proceeds via intramolecular nucleophilic attack of the Gly backbone amide nitrogen on the Asn side chain carbonyl carbon, followed by cyclization to a five-membered succinimide intermediate, deamidation and subsequent hydrolysis to yield both β- and α-aspartic acid products (Figure 1) [14].

The overall transformation is commonly described by a multistep kinetic scheme, where succinimide formation from Asn constitutes the rate-determining step characterized by the reaction rate coefficient *k*_1_ [14]:

(1) $$Asn\overset{k_{1}}{\to }Suc$$

(2) $$Suc\begin{array}{c} k_{2}\\ \begin{array}{c} \to \\ \leftarrow \end{array}\\ k_{-2} \end{array}\beta -Asp$$

(3) $$Suc\begin{array}{c} k_{3}\\ \begin{array}{c} \to \\ \leftarrow \end{array}\\ k_{-3} \end{array}\alpha -Asp$$

Although this kinetic framework is well established, the detailed nature of the proton-transfer events associated with succinimide formation remains incompletely understood. In particular, increasing evidence from proton-transfer and proton-coupled electron transfer (PCET) reactions suggests that nuclear quantum effects, including proton tunneling, can contribute to reaction rates. While the occurrence of tunneling in enzymatic reactions is not disputed, its mechanistic importance for enzymatic rate enhancement has been the goal of several investigations. In particular, there is ongoing debate as to whether protein dynamics directly facilitate tunneling or whether the dominant catalytic effect arises from electrostatic preorganization of the active site [15,16,17,18,19,20]. While such effects have been extensively investigated in enzymatic systems, their role in spontaneous peptide backbone rearrangements remains largely unexplored.

Hydrogen/deuterium (H/D) substitution in experiments leads to measurable changes in reaction rates and can be used to obtain data about kinetic isotope effect (*KIE*) value, as well as impact on activation energy (*E*_a_) and Arrhenius pre-exponential factor (A). These values provide a sensitive probe for distinguishing classical over-the-barrier processes from mechanisms involving quantum mechanical contributions to proton transfer, if the investigated system is close to classical limits, as is the case for the reaction observed herein. However, for calculations beyond the harmonic approximation, path integration with inclusion of thermal averaging would be the appropriate method [21,22,23]. The *KIE* can be calculated as the ratio of the two reaction rate coefficients:

(4) $$KIE=\frac{k_{1}(H)}{k_{1}(D)}$$

where *k*_1_(H) and *k*_1_(D) are the rate coefficients of the molecules containing the proton (protium) or deuterium isotope, respectively [24]. As Equation (1) can be considered as an elementary unimolecular reaction, it is reasonable to use the Arrhenius formula to describe its temperature dependence. Substituting the Arrhenius expressions for the rate coefficients into Equation (4) yields an expression from which the activation energy difference (Δ*E*_a_) and the pre-exponential factor ratio $ln\frac{A(H)}{A(D)}$ can be determined. Plotting the ln(*KIE*) against the $\frac{1}{T}$ values, a straight line can be obtained, the slope of which represents Δ*E*_a_ ($E_{a}\left(D\right)-E_{a}(H)$), and the intercept represents the $ln\frac{A(H)}{A(D)}$ value [21].

Importantly, isotope substitution is employed here as a mechanistic perturbation rather than a biological mimic, allowing direct insight into the underlying potential energy surface. The propensity for proton tunneling is strongly influenced by conformational dynamics that modulate donor–acceptor distances, a factor known to vary significantly across peptide sequence and local structure [25]. Here, we investigate the rate-determining succinimide formation step in NG isomerization using a combined computational and experimental approach. Density functional theory (DFT) and intrinsic reaction coordinate (IRC) calculations and analysis were performed on a deuterated Ac-Asn-NH-CH_3_ + H_2_O model system and compared with previously reported protonated pathways. In parallel, NG isomerization was examined experimentally under deuterated conditions and reduced temperatures using quantitative NMR spectroscopy, enabling determination of rate constants, half-lives, activation energies, kinetic isotope effects and impact on Arrhenius pre-exponential factors. By combining structural, energetic, and kinetic analyses across conformationally flexible (Ac-NGAA-NH_2_) and conformationally restricted (Ac-NGEA-NH_2_) model peptides, we aim to elucidate how local structural constraints modulate proton-transfer dynamics and to assess whether nuclear quantum effects contribute to spontaneous peptide backbone rearrangements relevant to protein aging processes.

## 2. Materials and Methods

### 2.1. QM Calculations

To investigate the effect of proton–deuterium exchange on the reaction mechanism of Ac-Asn-NH-CH_3_ + H_2_O, IRC path and calculations were completed at the B3LYP/6-31+G(d,p) level of theory in a vacuum at 298.15 K. We performed deuteration at every proton position that can get substituted when dissolved in D_2_O for laboratory experiments. The endpoint and the transition state optimizations, frequency and DFT calculations were completed at the B3LYP/6-311++G(d,p) level of theory in a vacuum (Note S1 and Tables S1–S8 within Supplementary Data S2). All were completed with the Gaussian 09 B01 and the Gaussian 16 C01 software packages. The protonated calculations were described in our previous article [14]. QM structures were visualized with the open-source Jmol 3D chemical structure viewer (http://www.jmol.org/).

### 2.2. Peptide Synthesis and Purification

The preparation of Ac-NGEA-NH_2_ was carried out by solid-phase peptide synthesis; for this, the Fmoc/tert-butyl strategy was used with Rink-amide MBHA resin, as in previous articles [13,14]. Peptide acetylation, and purification with a reversed-phase HPLC was also performed in a similar manner as previously described [13,14]. After the purification, the peptide was identified by ESI-MS and NMR spectroscopy. The Ac-NGAA-NH_2_ peptide was obtained from Gábor Mező and Kata Enyedi.

### 2.3. NMR Experiments

Time-dependent 1D ^1^H-NMR measurements (zgesgp) were carried out with a Bruker AVANCE-III 700 MHz instrument (Bruker, Billerica, MA, USA) (at Eötvös Loránd University) to follow the isomerization as we described in the Note S1 within Supplementary File S1 and previously [14]. Before the sample preparation, the peptides and the sodium phosphate buffer were twice lyophilized and resolved in D_2_O for the deuterated samples. The experiments were carried out at 28, 37 and 55 °C for protonated and deuterated Ac-NGAA-NH_2_ samples, and at 37 and 55 °C for protonated and deuterated Ac-NGEA-NH_2_ samples. The pH was adjusted to 7.4 for the protonated samples and to 7.0 for the deuterated samples, taking into account the effect of proton–deuterium exchange [14,26].

Only the deuterated measurements were performed in the current study, the protonated results were reported in our previous article [14]. The assignment of the deuterated measurements (Note S1–S3 and Figure S1–S6 within Supplementary Data S1) shows a high correspondence (Δδ_max_ < 2 Hz) for both the Ac-NGAA-NH_2_ and Ac-NGEA-NH_2_ samples compared to their protonated counterparts [14,27,28].

### 2.4. Determination of Kinetic Parameters

Based on the integral values of the relevant NMR signals, kinetic parameters were determined using the COPASI software application, version 4.27 (http://copasi.org/). The estimated parameters together with their standard deviations were the rate coefficients *k*_1_, *k*_2_, and *k*_3_, and the equilibrium constants *K*_2_ and *K*_3_. The rate coefficients *k*–_2_ and *k*–_3_ were calculated from $K_{2}=\frac{k_{2}}{k_{-2}}$ and $K_{3}=\frac{k_{3}}{k_{-3}}$ using the error propagation formula including the estimated covariances to calculate their standard deviations. All errors reported are half-widths of 95% confidence intervals based on the Student distribution of the estimated values.

The half-lives (*t*_1/2_) of the overall isomerization reactions were determined from *k*_1_ values using the following relation:

(5) $$t_{1/2}=\frac{ln2}{k_{1}}$$

Furthermore, the *KIE* values were calculated using the above-mentioned equation (Equation (4)). From the temperature dependence of the obtained *KIE* values, the ratio of Arrhenius pre-exponential factors and difference in activation energies (Δ*E*_a_) can be obtained using the following equations:

(6) $$\ln\left(KIE\right)=ln\frac{A(H)}{A(D)}+\frac{E_{a}\left(D\right)-E_{a}(H)}{R}\cdot \frac{1}{T}$$

(7) $$∆E_{a}=E_{a}\left(D\right)-E_{a}(H)$$

For comparison, Δ*E*_a_ values were determined from QM calculations with the following equations:

(8) $$∆H^{‡}=H_{transition\;state}-H_{reactant\;complex}$$

(9) $$E_{a}=∆H^{‡}+2RT$$

where *H* is the sum of electronic and thermal enthalpies (from Gaussian frequency calculation and energy optimalization output files).

## 3. Results and Discussion

### 3.1. QM Computational Results

We have previously examined the first major step of NG isomerization in detail in terms of changes in structural parameters, charge distribution and natural bond orbitals, in order to make conclusions regarding the driving force of the reaction. Based on our previous results, the first step of the NG isomerization reaction—which is also the rate-determining step for the overall isomerization, i.e., the formation of the succinimide ring—involves the transfer of protons, the transition state formation, the ring closure and the irreversible NH_3_ release microsteps [14]. To identify any potential differences or similarities with the previously identified reaction pathway and structural rearrangement, we performed deuteration of the Ac-Asn-NH-CH_3_ + H_2_O molecules at every proton position that can get substituted when dissolved in D_2_O for laboratory experiments. This H-D exchange involved the four protons bonded to the three nitrogen atoms and the two protons of the water molecule. The endpoint and the transition state optimizations, frequency and DFT calculations were completed at the B3LYP/6-311++G(d,p) level of theory in a vacuum at 298.15 K.

**Figure 2:**
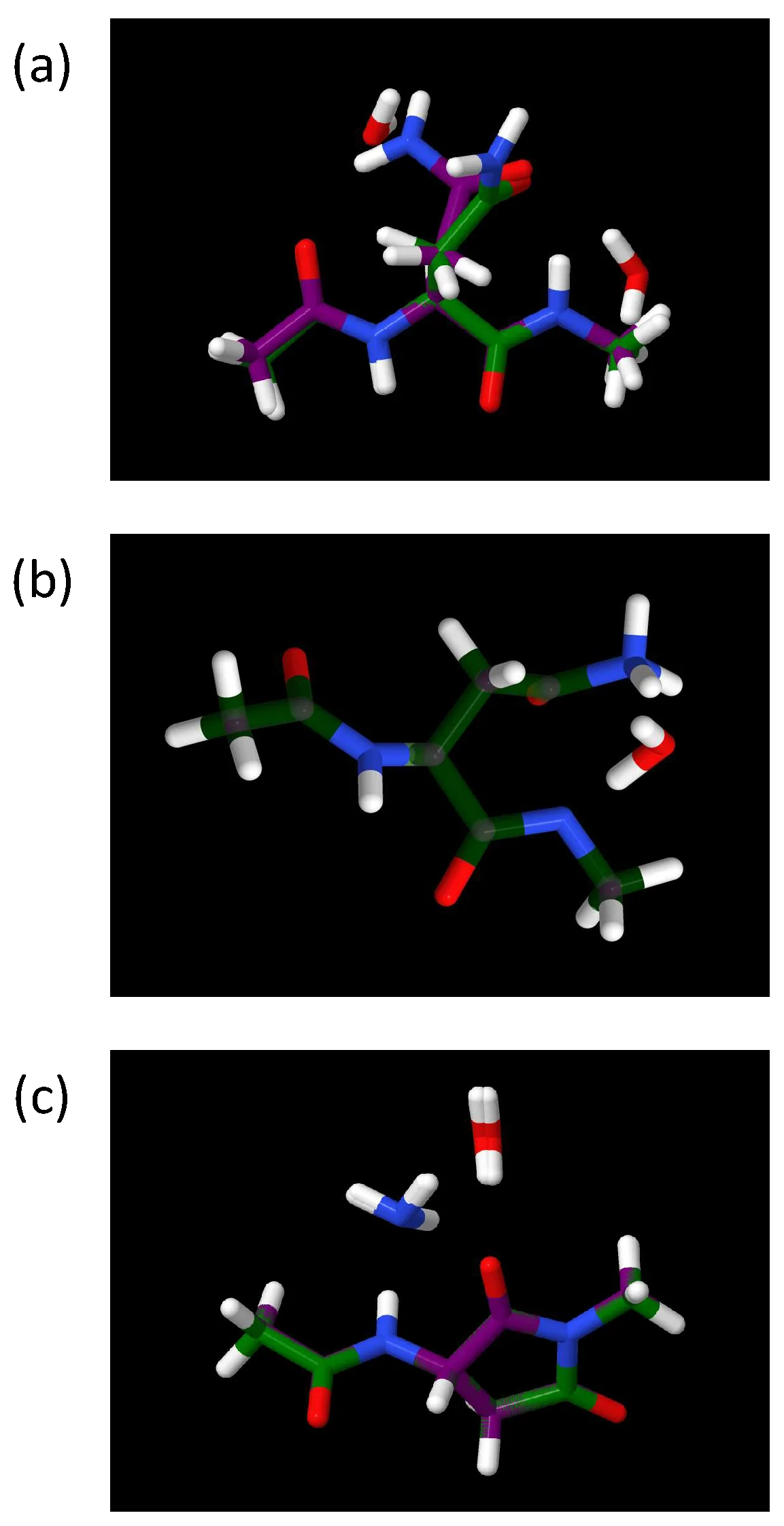
Structural comparison of the protonated [14] (green carbon atoms) and deuterated (purple carbon atoms) molecular structures. Comparative illustration of (**a**) the optimized open forms, (**b**) the transition states, and (**c**) the optimized succinimide structures calculated at the B3LYP/6-311++G(d,p) level of theory in a vacuum. All three pairs show a high degree of structural similarity.

For the structural comparison (Figure 2 and Table 1, and Tables S4, S6 and S8 within Supplementary Data S2), we used 4 + 1 angle values and 1 distance value, namely the *ϕ*, *ψ*, *χ*_1_, and *χ*_2_ dihedral angles as well as the Bürgi–Dunitz angle (N_Gly_-C^γ^_Asn_-O^δ^_Asn_, *θ*(BD)) and distance (N_Gly_-C^γ^_Asn_, *d*(BD)), which are indicators of the successful nucleophilic attack of the Gly nitrogen on the carbonyl carbon atom in the Asn side chain [29,30].

The structural comparison of the deuterated and the protonated molecules shows high similarity. Comparing the dihedral angles, we found deviations of less than 0.5% in all cases except for three. In these three cases, greater deviations are found between the open forms for the dihedral angles *ψ*, *χ*_1_, and *χ*_2_, which means an 18.6°, 30.98° and 100.58° difference. This suggests that there is a different orientation in the side chains, but the backbones still show a great similarity. The Bürgi–Dunitz angle in the deuterated molecule changes from an initial 67.45° to 109.78° as the molecule reaches a transition state and settles at 124.20° in the succinimide form, which is almost equal to the protonated case, where these values are 66.50°, 109.78° and 124.20°, respectively. The Bürgi–Dunitz distance decreases from an initial value of 3.38 Å to 2.64 Å in the transition state, and finally to 1.40 Å upon reaching the succinimide structure, which is again similar to the protonated case, where these values are 3.21 Å, 2.64 Å and 1.40 Å, respectively. The 109.78° Bürgi–Dunitz angle and the 2.64 Å bond length observed in the transition state also fulfill the conditions necessary for ring closure, as they are close to the ideal bond angle (109.5°) and bond length (3.25 Å) for a nucleophilic attack on the carbonyl group [29,30].

For further comparison, we calculated activation energies (*E*_a_) and the difference in activation energies (Δ*E*_a_) from the optimized open forms and the transition states (Table 2 and Tables S4, S6 and S8 within Supplementary Data S2).

The activation energies of the protonated and deuterated QM systems were found to be 193.17 and 203.64 kJ mol^−1^, respectively, which yields a difference of 10.47 kJ mol^−1^.

**Figure 3:**
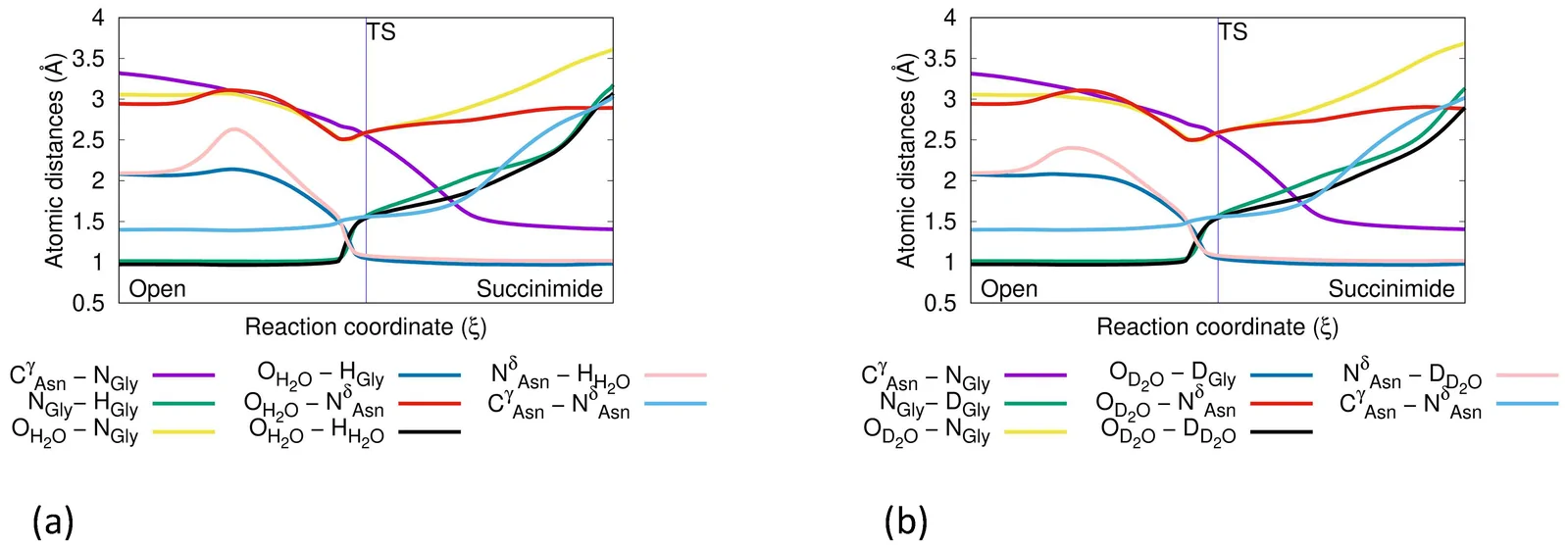
Changes in the interatomic distances between the atoms involved in the succinimide ring formation from the open form along the reaction coordinate. Changes in atomic distances (**a**) when they were present as protons (this case was already discussed in great detail in our previous article [14]) and (**b**) when the protons were replaced by deuterium atoms at every proton position that can get substituted when dissolved in D_2_O for laboratory experiments. All changes in interatomic distances show a high degree of similarity between the protonated and deuterated QM systems.

In addition to the Bürgi–Dunitz distance described above, we were also able to compare the distances between other atoms in the reaction center of the deuterated system (Figure 3) as we did previously with the protonated one.

The two diagrams showing distance pairs also suggest, in agreement with the above findings, that there is no significant difference between the deuterated and protonated classical reaction pathways. The same pairs of atoms that change together are present, demonstrating how the N_Gly_-D_Gly_, O_D2O_-D_D2O_ and C^γ^_Asn_-N^δ^_Asn_ (Figure 3a—green, black and light blue) bonds present in the initial phase of the reaction decompose as the reaction progresses, and how bonds O_D2O_-D_Gly_, N^δ^_Asn_-D_D2O_ and C^γ^_Asn_-N_Gly_ (Figure 3a—blue, light pink and purple) form between other pairs of atoms.

### 3.2. Kinetic Analysis Based on NMR Experiments

In our experiments, we followed the NG isomerization of both Ac-NGAA-NH_2_ and Ac-NGEA-NH_2_ peptides in their deuterated form, performed a detailed kinetic analysis and compared the obtained parameters to our previous results on protonated samples [14]. As mentioned above, NG isomerization can be described by five reaction rate coefficients (Equations (1)–(3); *k*_1_, *k*_2_, *k*_−2_, *k*_3_ and *k*_−3_). The comparison of the *k*_1_, *k*_2_ and *k*_3_ reaction rate coefficients (Table 3) of the deuterated experiments shows that the rate-determining step is the deamidation and succinimide ring closure, as the *k*_1_ values are smaller than the *k*_2_ and *k*_3_ values also under deuterated conditions, similarly to the protonated experiments [14]. This alignment, alongside the QM computational results for the rate-determining step, further strengthens the idea that the isomerization proceeds via a similar overall reaction pathway for both the protonated and the deuterated samples, if the classical reaction mechanism is considered. Furthermore, the *k*_1_ reaction rate coefficient values, in agreement with previous literature results [13,14], show in both protonated and deuterated experiments that lowering the temperature decelerates the reaction (Table 3).

After the irreversible rate-determining step, the isomerization becomes complete with two equilibrated processes (Equations (2) and (3)); therefore, we can establish *k*_−2_ and *k*_−3_ values for the conversion back to succinimide alongside the *k*_2_ and *k*_3_ values. A comparison of the equilibrium constants $K_{2}=\frac{k_{2}}{k_{-2}}$ and $K_{3}=\frac{k_{3}}{k_{-3}}$ suggests that, under the experimental conditions examined in this article, K_2_ is generally higher, with the β-Asp product being formed in greater proportions than the α-Asp product under all circumstances, even in the deuterated case, just as we observed with the protonated version, further supporting the similarity in the reaction outcomes.

In addition to the tetrapeptide containing alanine with a neutral side chain at the third amino acid position, we also performed deuterated experiments with a peptide containing glutamic acid with a negatively charged side chain at the same amino acid position. As we have previously established [14], the reaction rate of the Ac-NGEA-NH_2_ peptide containing glutamic acid is significantly lower than that of the Ac-NGAA-NH_2_ peptide containing neutral alanine under all experimental conditions we have tested. Furthermore, within the temperature range we examined, it is true that the reaction rate of the glutamic-acid-containing peptide decreases as the temperature decreases. According to these, the previous [14] and current experimental results support the fact that proton–deuterium exchange has no effect on the trends observed in such experimental outcomes. However, deuteration leads to slower reactions with higher half-life values (*t*_1/2_) in all cases.

From the *k*_1_ values of the reactions, we obtained *KIE* values for each peptide and temperature. In the case of the Ac-NGAA-NH_2_ peptide, the calculated *KIE* values show that at 55 °C, where the molecules are more likely to have sufficient energy to overcome the potential energy barrier, a value of 5.02 is obtained. As the temperature, and therefore the energy, decreases, the experiments result in higher *KIE* values over the investigated temperature range: 6.25 at 37 °C and 6.88 at 28 °C. Reactions whose primary *KIE* value exceeds 6–10 can no longer be explained solely by the difference in zero-point energies between lighter and heavier isotopes—and thus by a classical reaction mechanism—but instead indicate the presence of the tunneling effect [21,31]. Comparing this with the observed *KIE* values, which show an increasing trend as the temperature decreases, it can be concluded that the reaction shifts toward the tunneling mechanism within the 55–28 C° range. In contrast, for the Ac-NGEA-NH_2_ peptide, we observed much lower *KIE* values with almost no temperature dependence, which are 2.37 at 55 °C and 2.64 at 37 °C.

**Figure 4:**
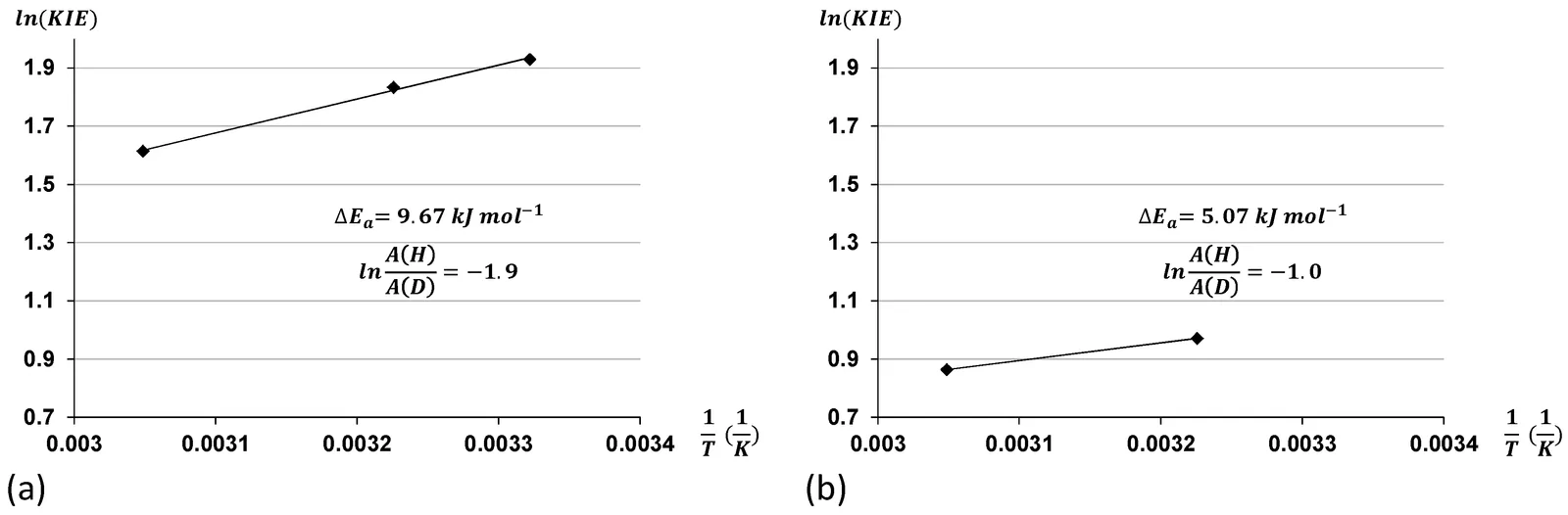
Temperature dependence of *KIE* values. The ln(*KIE*) values obtained for (**a**) Ac-NGAA-NH_2_ and (**b**) Ac-NGEA-NH_2_ peptides are shown as a function of 1/*T*. Numerical values of the difference in activation energies (Δ*E*_a_) between protonated and deuterated forms (slope) and the intercept $ln\frac{A(H)}{A(D)}$, the ratio of Arrhenius pre-exponential factors, are annotated in the figure. Both values are sensitive indicators of tunneling.

The temperature dependence of *KIE* values can be evaluated through plotting ln(*KIE*) against 1/*T* (Figure 4). The fitted line is expected to be linear, where the slope gives the difference in activation energies (Δ*E*_a_) between protonated and deuterated forms, and the intercept gives $ln\frac{A(H)}{A(D)}$, the ratio of Arrhenius pre-exponential factors, both being sensitive indicators of tunneling. In this sense, a Δ*E*_a_ value greater than 5.86 kJ mol^−1^ (1.4 kcal mol^−1^) and an $ln\frac{A(H)}{A(D)}$ value of less than −0.7 are both evaluated as being traditional markers of tunneling effects [21].

In the case of the Ac-NGEA-NH_2_ peptide, the Δ*E*_a_ value is 5.07 kJ mol^−1^, which is less than the tunneling limit, indicating a classical mechanism. The low *KIE* values reported above (Table 3) support the same mechanism, while the $ln\frac{A(H)}{A(D)}$ value of −1.0 exceeds the tunneling limit, but only slightly. In contrast, the Ac-NGAA-NH_2_ peptide shows a Δ*E*_a_ value of 9.67 kJ mol^−1^, clearly indicating tunneling contributions to the mechanism, as well as the $ln\frac{A(H)}{A(D)}$ value of −1.9. As mentioned above, the *KIE* values of Ac-NGAA-NH_2_ (Table 3) also approached the tunneling limits, indicating a mixed classical–tunneling mechanism, especially at lower temperatures, where the classical over-the-barrier mechanism is less likely to occur.

The main difference between the Ac-NGEA-NH_2_ and Ac-NGAA-NH_2_ peptides lies in their structural flexibility. As we previously proposed, the structure of the Ac-NGEA-NH_2_ peptide is more constrained, as it may adopt a β-turn-like conformer. This conformer disfavors succinimide formation as the Bürgi–Dunitz distance and angle are far from the ideal [14]. This explains our observation about the absence of tunneling contribution to the reaction mechanism in the case of Ac-NGEA-NH_2_, since quantum tunneling requires close spatial proximity. In contrast, Ac-NGAA-NH_2_ and Ac-Asn-NH-CH_3_ peptides are flexible enough to sample multiple conformational states, thereby providing opportunity for being in spatial proximity, needed for succinimide formation, and enabling both classical mechanism and tunneling contribution. This is consistent with the fact that the experimentally obtained Δ*E*_a_ value of 9.67 kJ mol^−1^ (Ac-NGAA-NH_2_) is very close to the computational value of 10.47 kJ mol^−1^ (Ac-Asn-NH-CH_3_ + H_2_O), indicating that the experimental and computational results are in good agreement for the two investigated flexible systems. It can be concluded that the nature of the succinimide formation is a mixed classical–tunneling mechanism, if the system is structurally not constrained.

## 4. Conclusions

Combined quantum chemical calculations and quantitative NMR kinetic analyses demonstrate that proton–deuterium exchange does not alter the fundamental classical reaction pathway of NG isomerization. Structural comparisons of the protonated and deuterated QM systems revealed highly similar conformational evolution along the reaction coordinate, including comparable Bürgi–Dunitz geometries, transition-state arrangements, and reaction-center distance changes associated with succinimide ring formation. Consistent with these observations, the experimentally determined kinetic profiles retained the same overall topology under both protonated and deuterated conditions. According to this, succinimide formation remains the rate-determining step of the isomerization process after H/D isotope exchange.

Despite the preservation of the classical pathway, deuteration produced substantial kinetic effects. Importantly, the computational results indicate that the observed kinetic isotope effects originate primarily from differences in proton-transfer energetics rather than from major structural alteration of the reaction pathway, which enables the evaluation of kinetic isotope effects (*KIE*). Based on the results derived from NMR experiments of the Ac-NGAA-NH_2_ peptide, the determined *KIE*, Δ*E*_a_ and $ln\frac{A(H)}{A(D)}$ values together support the presence of a significant tunneling contribution during the rate-determining succinimide-forming step. In particular, the progressive increase in the *KIE* from 5.02 at 55 °C to 6.88 at 28 °C is consistent with a mixed classical–tunneling mechanism, in which the contribution of tunneling becomes more probable at lower temperatures. It is noteworthy that this temperature-dependent change in *KIE* is reflected in the Δ*E*_a_ value of 9.67 kJ mol^−1^, which is also indicative of tunneling and in close agreement with the computational Δ*E*_a_ value of 10.47 kJ mol^−1^.

Another notable outcome of this study is the strong contrast between the experimentally investigated Ac-NGAA-NH_2_ and Ac-NGEA-NH_2_ peptides, which represents conformationally more flexible and restricted systems, respectively. While the Ac-NGAA-NH_2_ system displayed a mixed classical–tunneling mechanism, for the Ac-NGEA-NH_2_ peptide, the determined *KIE*, Δ*E*_a_ and $ln\frac{A(H)}{A(D)}$ values together suggest that there is no relevant tunneling contribution in the succinimide-forming step. These findings suggest that conformational accessibility may play a critical role in regulating tunneling probability in spontaneous peptide isomerization reactions.

The present results therefore extend the relevance of tunneling phenomena beyond well-studied enzyme-catalyzed proton-transfer reactions to spontaneous peptide backbone rearrangements. Since NG motifs are widespread in long-lived proteins susceptible to deamidation and isomerization, these findings raise the possibility that tunneling may contribute to nonenzymatic protein aging pathways under physiologically relevant conditions. Further investigation of larger peptide and protein systems may provide valuable insight into the role of quantum mechanical effects in spontaneous post-translational modifications and long-term protein stability.

## Figures and Tables

**Table 1:** Structural parameters of the open, transition state and succinimide ring structures. Comparison of the structural parameters of protonated [14] and deuterated QM systems (shown in Figure 2 and Tables S4, S6 and S8 within Supplementary Data S2), namely dihedral angles (*ϕ*, *ψ*, *χ*_1_, and *χ*_2_) as well as the Bürgi–Dunitz angle (N_Gly_-C^γ^_Asn_-O^δ^_Asn_, *θ*(BD)) and distance (N_Gly_-C^γ^_Asn_, *d*(BD)).

	Protonated Open	Deuterated Open	Protonated Transition State	Deuterated Transition State	Protonated Succinimide	Deuterated Succinimide
*ϕ*/°	−166.45	−167.07	−171.61	−171.61	−128.64	−128.70
*ψ*/°	162.92	−178.48	−140.95	−140.95	−141.97	−141.94
*χ*_1_/°	−165.63	−134.65	−176.45	−176.45	140.71	140.68
*χ*_2/_°	−165.98	93.44	−134.04	−134.04	−59.61	−59.60
*θ*(BD)/°	66.50	67.45	109.78	109.78	124.20	124.20
*d*(BD)/Å	3.21	3.38	2.64	2.64	1.40	1.40

**Table 2:** Energy values of the protonated [14] and deuterated QM systems calculated at the B3LYP/6-311++G(d,p) level of theory in a vacuum at 298.15 K. *H* values are the sum of electronic and thermal enthalpies (from Gaussian frequency calculation and energy optimalization output files) for open, transition state (TS) and succinimide forms. From these values, the activation energies (*E*_a_) and the difference of activation energies (Δ*E*_a_) were calculated.

	Protonated	Deuterated
*H* (open)/Hartree	−740.977393	−741.000365
*H* (TS)/Hartree	−740.905707	−740.924692
*H* (succinimide)/Hartree	−740.988360	−741.007298
Δ*H*^‡^ (open-TS)/kJ mol^−1^	188.21	198.68
*E*_a_ (open-TS)/kJ mol^−1^	193.17	203.64
Δ*E*_a_ (open-TS)/kJ mol^−1^	10.47	

**Table 3:** Reaction rate coefficients and half-life values of protonated and deuterated peptides and their corresponding kinetic isotope effect (*KIE*) values. For protonated [14] and deuterated Ac-NGAA-NH_2_ and Ac-NGEA-NH_2_ peptides, the isomerization reaction was observed by NMR at different temperatures and reaction rate coefficients (Equations (1)–(3); *k*_1_, *k*_2_, *k*_−2_, *k*_3_ and *k*_−3_), and half-life values (Equation (5); *t*_1/2_) were determined. At each temperature, kinetic isotope effect (*KIE*) values were calculated according to Equation (4). Error values refer to the half-widths of their 95% confidence intervals. The abbreviation ‘n. d.’ stands for ‘not determined’.

Peptide	*T*, °C		*k*_1_, h^−1^	*k*_2_, h^−1^	*k*_−2_, h^−1^	*k*_3_, h^−1^	*k*_−3_, h^−1^	*t*_1/2_, h	*KIE*
Protonated -NGAA-	55	value	0.2192	5.211	0.0938	0.68	0.0346	3.2	5.02
		error	0.0039	0.163	0.0044	0.08	0.0088		
Deuterated -NGAA-	55	value	0.0436	1.356	0.012	0.4106	0.0126	15.9	
		error	0.00041	0.019	0.00044	0.0073	0.00073		
Protonated -NGAA-	37	value	0.027	0.714	n. d.	n. d.	n. d.	25.7	6.25
		error	0.008	0.32	n. d.	n. d.	n. d.		
Deuterated -NGAA-	37	value	0.0043	0.1835	0.0009	0.0585	0.00147	160.5	
		error	0.00011	0.0052	0.00011	0.0022	0.00016		
Protonated -NGAA-	28	value	0.0047	0.120	n. d.	0.055	0.017	147.5	6.88
		error	0.00047	0.023	n. d.	0.033	0.011		
Deuterated -NGAA-	28	value	0.00068	0.0194	0.00001	0.01003	0.0007	1014.0	
		error	0.000026	0.0068	n. d.	0.0052	n. d.		
Protonated -NGEA-	55	value	0.057	2.03	0.043	0.385	n. d.	12.2	2.37
		error	0.007	0.44	0.044	0.24	n. d.		
Deuterated -NGEA-	55	value	0.024	1.0239	0.0041	0.2373	0.0039	28.8	
		error	0.0016	0.0821	0.00081	0.0186	0.00105		
Protonated -NGEA-	37	value	0.0075	0.327	0.0052	0.058	n. d.	92.4	2.64
		error	0.0003	0.022	0.0023	0.005	n. d.		
Deuterated -NGEA-	37	value	0.00284	0.1987	0.0046	0.0342	0.0028	244.1	
		error	0.000021	0.0025	0.00021	0.0008	0.00038		

## Data Availability

The authors declare that the data supporting the findings of this study are available within the paper and its Supplementary File S1, Supplementary Data S1–S2 files. All other relevant data are available from the corresponding author upon reasonable request.

## Supplementary Materials

The following supporting information can be downloaded at: https://www.mdpi.com/article/10.3390/biom16091266/s1, Supplementary File S1. Note S1: 1H-NMR spectra recording, processing, and analysis procedures. Figure S1: An example of 1D ^1^H-NMR spectrum of deuterated Ac-NGEA-NH_2_ peptide. Note S2: Figures S2–S6: Diagrams of the measured and fitted relative concentrations determined by the parameter estimation process, measured at 55, 37 and 28°C for Ac-NGAA-NH_2_ peptide and at 55 and 37°C for Ac-NGEA-NH_2_ peptide. Supplementary Data S1. ^1^H-NMR resonance assignment, coupling patterns and coupling constants. Note S1: Technical details of NMR measurements. Note S2: Signal assignment and coupling constants of deuterated Ac-NGAA-NH_2_ peptide and the products of its isomerization reaction (Ac-αDGAA-NH_2_ and Ac-βDGAA-NH_2_). Figure S1: 1D ^1^H-NMR spectrum of deuterated Ac-NGAA-NH_2_ peptide. Figure S2: 1D ^1^H-NMR spectrum of the products of the isomerization reaction of deuterated Ac NGAA-NH_2_ (Ac-αDGAA-NH_2_ and Ac-βDGAA-NH_2_). Figure S3: Enlargements of 1D ^1^H-NMR spectra of deuterated Ac-NGAA-NH_2_, and the products of its isomerization reaction (Ac-αDGAA-NH_2_ and Ac-βDGAA-NH_2_). Note S3: Signal assignment and coupling constants of deuterated Ac-NGEA-NH_2_ peptide and the products of its isomerization reaction (Ac-αDGEA-NH_2_ and Ac-βDGEA-NH_2_). Figure S4: 1D ^1^H-NMR spectrum of deuterated Ac-NGEA-NH_2_ peptide. Figure S5: 1D ^1^H-NMR spectrum of deuterated Ac NGEA-NH_2_ and the products of its isomerization reaction (Ac-αDGEA-NH_2_ and Ac-βDGEA-NH_2_). Figure S6: Enlargements of 1D ^1^H-NMR spectra of deuterated Ac-NGEA-NH_2_, and the products of its isomerization reaction (Ac-αDGEA-NH_2_ and Ac-βDGEA-NH_2_). Supplementary Data S2. Input parameters, coordinates and absolute energies of the structures used in IRC path calculations, structure optimizations and analysis. Note S1: Input parameters for IRC path calculations. Table S1: Cartesian coordinates and input parameters of open form of the deuterated system. Table S2: Cartesian coordinates and energy value of iteration step 43 of the open form of the deuterated system. Table S3: Cartesian coordinates and input parameters of iteration step 43 of the open form of the deuterated system. Table S4: Cartesian coordinates and energy values of the optimized open form of the deuterated system. Table S5: Cartesian coordinates and input parameters of transition state of the deuterated system. Table S6: Cartesian coordinates and energy values of the transition state of the deuterated system. Table S7: Cartesian coordinates and input parameters of succinimide of the deuterated system. Table S8: Cartesian coordinates and energy values of the optimized succinimide of the deuterated system.

## Abbreviations

BD	Bürgi–Dunitz
DFT	Density functional theory
ESI-MS	Electrospray ionization mass spectrometry
H/D	Hydrogen/deuterium
HPLC	High-performance liquid chromatography
IRC	Intrinsic reaction coordinate
*KIE*	Kinetic isotope effect
NG	Asn-Gly, asparagine-glycine
NMR	Nuclear magnetic resonance
PCET	Proton-coupled electron transfer
QM	Quantum mechanical
TS	Transition state
