# Supplementary material for: Impact of Structural Flexibility on Tunneling Contribution to the Reaction Mechanism of Spontaneous Succinimide Formation in Asn-Gly-Containing Peptides
Source: Biomolecules. 2026 Sep 1;16(9):1266. doi: 10.3390/biom16091266 (PMC13604283; doi:10.3390/biom16091266)
Supplement: Supplementary file 1 [file biomolecules-16-01266-s001.zip › Supplementary File S1.pdf]

Supplementary File S1

Impact of Structural Flexibility on Tunneling Contribution to the Reaction Mechanism of Spontaneous Succinimide Formation in Asn-Gly-Containing Peptides

Fruzsina Pilhál [1,2], Bianka Szalainé Ágoston [1,3], Imre Jákli [1,4], Ernő Keszei [5] and András Perczel [1,4,*]

1 F. Pilhál, Dr. B. Szalainé Ágoston, Dr. I. Jákli, Prof. A. Perczel
Laboratory of Structural Chemistry and Biology, Institute of Chemistry
ELTE Eötvös Loránd University
Pázmány Péter sétány. 1/A, 1117 Budapest, Hungary
* E-mail: A.P. perczel.andras@ttk.elte.hu

2 F. Pilhál
Hevesy György PhD School of Chemistry
ELTE Eötvös Loránd University
Pázmány Péter sétány. 1/A, 1117 Budapest, Hungary

3 B. Szalainé Ágoston
Present address: Faculty of Information Technology and Bionics
Pázmány Péter Catholic University
Práter utca 50/a, 1083 Budapest, Hungary

4 Dr. I. Jákli, Prof. A. Perczel
HUN-REN–ELTE Protein Modelling Research Group, Institute of Chemistry
ELTE Eötvös Loránd University
Pázmány Péter sétány. 1/A, 1117 Budapest, Hungary

5 Prof. E. Keszei
Department of Physical Chemistry and Chemical Kinetics Laboratory, Institute of Chemistry
ELTE Eötvös Loránd University
Pázmány Péter sétány. 1/A, 1117 Budapest, Hungary

Table of Contents

Supplementary Note S1 Note S1 ^{1}H-NMR spectra recording, processing, and analysis procedures

Supplementary Note S2 Note S2 Detailed kinetic analysis - Diagrams of the measured and fitted relative concentrations determined by the parameter estimation process

Supplementary Note S1 ^{1}H-NMR spectra recording, processing, and analysis procedures

1D ^{1}H-NMR spectra (Supplementary Figure S1) with zgesgp pulse program were measured with the following parameters: TD (time domain) = 16384, NS (number of scans) = 64, SW (spectral width) = 11 ppm (for deuterated Ac-NGAA-NH_2) and 12 ppm (for deuterated Ac-NGEA-NH_2), AQ (acquisition time) = 1.07 sec (for deuterated Ac-NGAA-NH_2) and 0.97 sec (for deuterated Ac-NGEA-NH_2), d1 (delay time) = 1.5 sec, LB (line broadening of exponential window function) = 0.30 Hz.

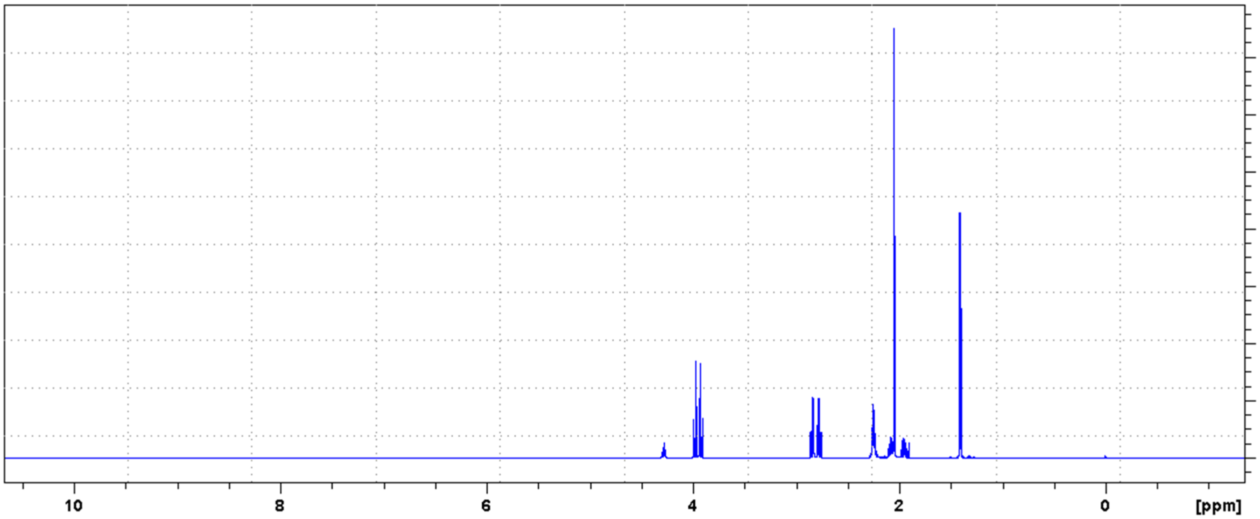

Supplementary Figure S1 **An 1D ^{1}H-NMR spectrum of deuterated Ac-NGEA-NH_2 peptide measured at pH=7.0 and 37 °C, 700 MHz.**

In all spectra the processing started with the *efp* command, then the 0th order correction was done manually for each spectra. After phasing, the DSS signal was calibrated to 0 ppm. By overlaying the spectra calibrated to 0 ppm, the integration limits (regions) for the peptide signals were also determined. The integration limits were determined manually for each measurement condition separately, and the resulting relative integral values and chemical shift values were collected. Then spectra with insufficient shimming or baseline errors were removed from the collected measurement data. The parameter estimation for the remaining data points were then performed using the Copasi software.

Supplementary Note S2 Detailed kinetic analysis - Diagrams of the measured and fitted relative concentrations determined by the parameter estimation process.

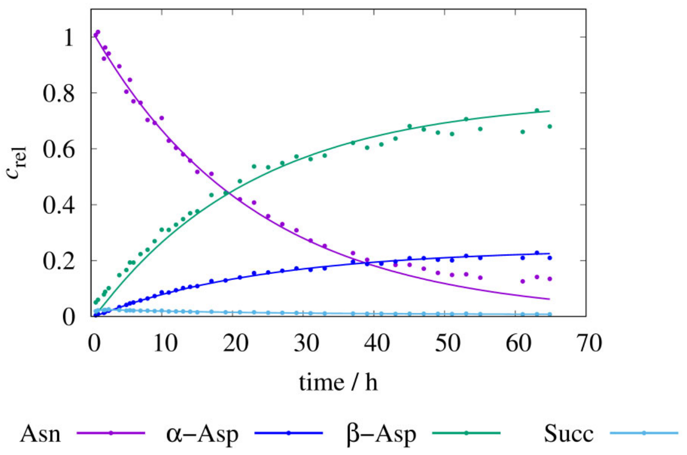

Supplementary Figure S2 **The measured and fitted time course of the deuterated Ac-NGAA-NH_2 isomerisation reaction at pH=7.0, 55 °C.** The decomposition of deuterated Ac-NGAA-NH_2 via isomerisation and the formation of the products β-Asp (β-DGAA) and α-Asp (α-DGAA), respectively (pH=7.0, T=55 °C), via the intermediate succinimide (Suc). The vertical axis is scaled with arbitrary units proportional to selected non-overlapping NMR signal integrals plotted as a function of the time.

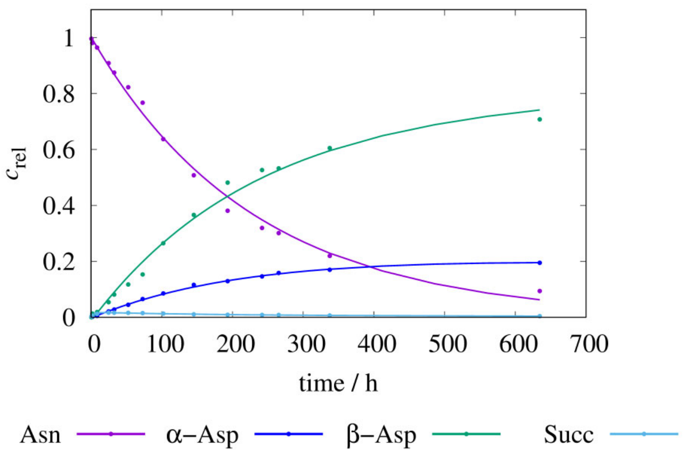

Supplementary Figure S3 **The measured and fitted time course of the deuterated Ac-NGAA-NH_2 isomerisation reaction at pH=7.0, 37 °C.** The decomposition of deuterated Ac-NGAA-NH_2 via isomerisation and the formation of the products β-Asp (β-DGAA) and α-Asp (α-DGAA), respectively (pH=7.0, T=37 °C), via the intermediate succinimide (Suc). The vertical axis is scaled with arbitrary units proportional to selected non-overlapping NMR signal integrals plotted as a function of the time.

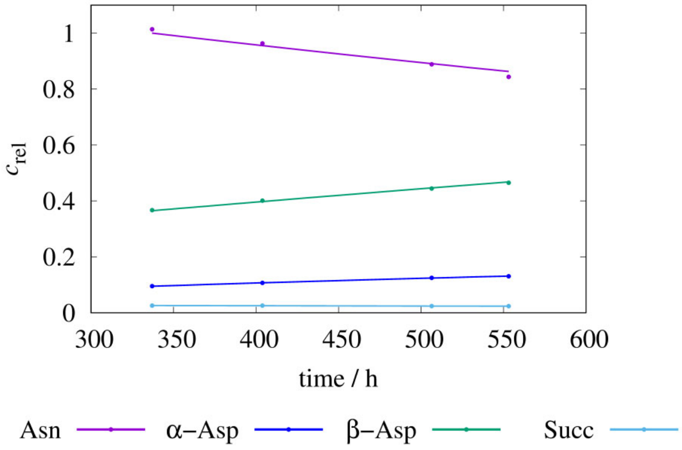

Supplementary Figure S4 **The measured and fitted time course of the deuterated Ac-NGAA-NH_2 isomerisation reaction at pH=7.0, 28 °C.** The decomposition of deuterated Ac-NGAA-NH_2 via isomerisation and the formation of the products β-Asp (β-DGAA) and α-Asp (α-DGAA), respectively (pH=7.0, T=28 °C), via the intermediate succinimide (Suc). The vertical axis is scaled with arbitrary units proportional to selected non-overlapping NMR signal integrals plotted as a function of the time.

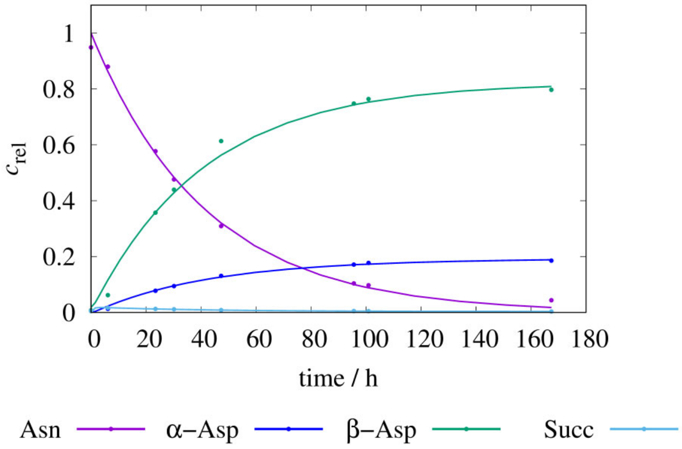

Supplementary Figure S5 **The measured and fitted time course of the deuterated Ac-NGEA-NH_2 isomerisation reaction at pH=7.0, 55 °C.** The decomposition of deuterated Ac-NGEA-NH_2 via isomerisation and the formation of the products β-Asp (β-DGEA) and α-Asp (α-DGEA), respectively (pH=7.0, T=55 °C), via the intermediate succinimide (Suc). The vertical axis is scaled with arbitrary units proportional to selected non-overlapping NMR signal integrals plotted as a function of the time.

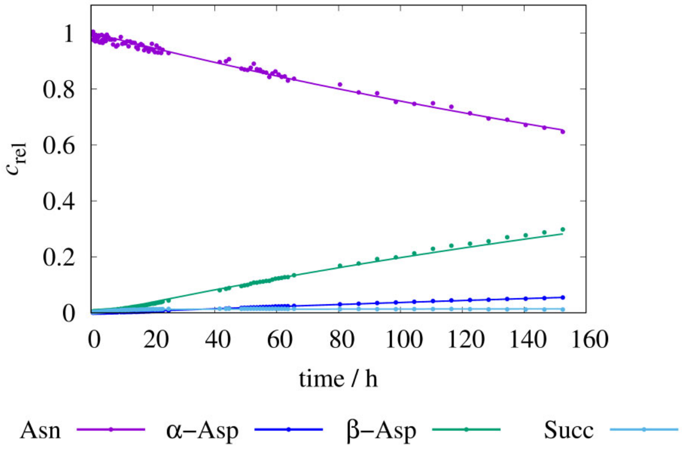

Supplementary Figure S6 **The measured and fitted time course of the deuterated Ac-NGEA-NH_2 isomerisation reaction at pH=7.0, 37 °C.** The decomposition of deuterated Ac-NGEA-NH_2 via isomerisation and the formation of the products β-Asp (β-DGEA) and α-Asp (α-DGEA), respectively (pH=7.0, T=37 °C), via the intermediate succinimide (Suc). The vertical axis is scaled with arbitrary units proportional to selected non-overlapping NMR signal integrals plotted as a function of the time.
