# Supplementary material for: Impact of Structural Flexibility on Tunneling Contribution to the Reaction Mechanism of Spontaneous Succinimide Formation in Asn-Gly-Containing Peptides
Source: Biomolecules. 2026 Sep 1;16(9):1266. doi: 10.3390/biom16091266 (PMC13604283; doi:10.3390/biom16091266)
Supplement: Supplementary file 1 [file biomolecules-16-01266-s001.zip › Supplementary Data S1.pdf]

Supplementary Data S1

^{1}H-NMR resonance assignment, coupling patterns and coupling constants

Impact of Structural Flexibility on Tunneling Contribution to the Reaction Mechanism of Spontaneous Succinimide Formation in Asn-Gly-Containing Peptides

Fruzsina Pilhál [1,2], Bianka Szalainé Ágoston [1,3], Imre Jákli [1,4], Ernő Keszei [5] and András Perczel [1,4,*]

1 F. Pilhál, Dr. B. Szalainé Ágoston, Dr. I. Jákli, Prof. A. Perczel
Laboratory of Structural Chemistry and Biology, Institute of Chemistry
ELTE Eötvös Loránd University
Pázmány Péter sétány. 1/A, 1117 Budapest, Hungary
* E-mail: A.P. perczel.andras@ttk.elte.hu

2 F. Pilhál
Hevesy György PhD School of Chemistry
ELTE Eötvös Loránd University
Pázmány Péter sétány. 1/A, 1117 Budapest, Hungary

3 B. Szalainé Ágoston
Present address: Faculty of Information Technology and Bionics
Pázmány Péter Catholic University
Práter utca 50/a, 1083 Budapest, Hungary

4 Dr. I. Jákli, Prof. A. Perczel
HUN-REN–ELTE Protein Modelling Research Group, Institute of Chemistry
ELTE Eötvös Loránd University
Pázmány Péter sétány. 1/A, 1117 Budapest, Hungary

5 Prof. E. Keszei
Department of Physical Chemistry and Chemical Kinetics Laboratory, Institute of Chemistry
ELTE Eötvös Loránd University
Pázmány Péter sétány. 1/A, 1117 Budapest, Hungary

Supplementary Data S1 Note S1: ^{1}H-NMR resonance assignment

NMR spectra were recorded on Bruker AVANCE-III 700 MHz NMR spectrometer at the Eötvös Loránd University, Budapest. Data processing was done using Bruker TopSpin 3.5 and 3.6 software versions. The assignment of the 1D ^{1}H-NMR spectra of deuterated Ac-NGAA-NH_2 and deuterated Ac-NGEA-NH_2 peptides at different temperatures, in 50 mM Na_2HPO_4 buffer was performed based on the assignment of their protonated counterparts and on typical J-coupling values as well as typical chemical shifts of the protons (δ) [1,2].

Supplementary Data S1 Note S2: Signal assignment and coupling constants of deuterated Ac-NGAA-NH_2 peptide and the products of its isomerization reaction (Ac-*α*DGAA-NH_2 and Ac-*β*DGAA-NH_2) by 1D ^{1}H-NMR spectra at 700 MHz, 37 °C, in 50 mM Na_2HPO_4 buffer in D_2O, with the pH adjusted to 7.0, taking into account the effect of proton-deuterium exchange[3] (corresponds to pH 7.4 in H_2O).

1: δ(DSS) = 0.0000 ppm (s)

2: Composite multiplet: δ = 1.3951 ppm (m) composed of ALA-HB signals of residues 3 and 4, of molecules containing only Asn.
3: Composite multiplet: δ = 1.3984 ppm (m) composed of ALA-HB signals of residues 3 and 4, of molecules containing either α-Asp or β-Asp.

4: δ(β-Asp-acetyl-H) = 2.0028 ppm (s)
5: δ(Suc-acetyl-H) = 2.0322 ppm (s)
6: δ(α-Asp-acetyl-H) = 2.0367 ppm (s)
7: δ(Asn-acetyl-H) = 2.0422 ppm (s)

8: δ(α-ASP-HB) = 2.6759 ppm (dd), 2.6237 ppm (dd), ^{2}J(α-ASP-HB2:HB3) = 16.09 Hz, ^{3}J(α-ASP-HB:HA) = 5.60, 7.72 Hz
9: δ(β-ASP-HB) = 2.8126 ppm (dd), 2.6599 ppm (dd), ^{2}J(β-ASP-HB2:HB3) = 15.12 Hz, ^{3}J(β-ASP-HB:HA) = 5.42 Hz, 8.28 Hz
10: δ(ASN-HB) = 2.8335 ppm (dd), 2.7593 ppm (dd), ^{2}J(ASN-HB2:HB3) = 15.81 Hz, ^{3}J(ASN-HB:HA) = 5.63 Hz, 7.81 Hz

11: δ(Suc-HB) = 3.2340 ppm (m).

12: Composite multiplet: δ = 3.9208 ppm (m), composed of GLY-HA signals of molecules containing either α-Asp or β-Asp.

13: δ(GLY-HA) = 3.9528 ppm (d), 3.9078 ppm (d), ^{2}J(GLY-HA2:HA3) = 16.91 Hz, signal of molecules containing Asn.

14: Composite multiplet: δ = 4.2931 ppm (m) composed of ALA-HA of residues 3 and 4, of molecules containing either Asn, α-Asp or β-Asp.

In the NH region no signals are expected due to proton-deuterium exchange. However, low intensity signals are observed due to incomplete deuteration.

ASN-HA signal is expected to be within the water suppression region, and accordingly cannot be seen in the spectrum.

Supplementary Data S1 Figure S1 1D ^{1}H-NMR spectrum of deuterated Ac-NGAA-NH_2 peptide measured at 700 MHz, 37 °C, in 50 mM Na_2HPO_4 buffer in D_2O, with the pH adjusted to 7.0, taking into account the effect of proton-deuterium exchange[3] (corresponds to pH 7.4 in H_2O).

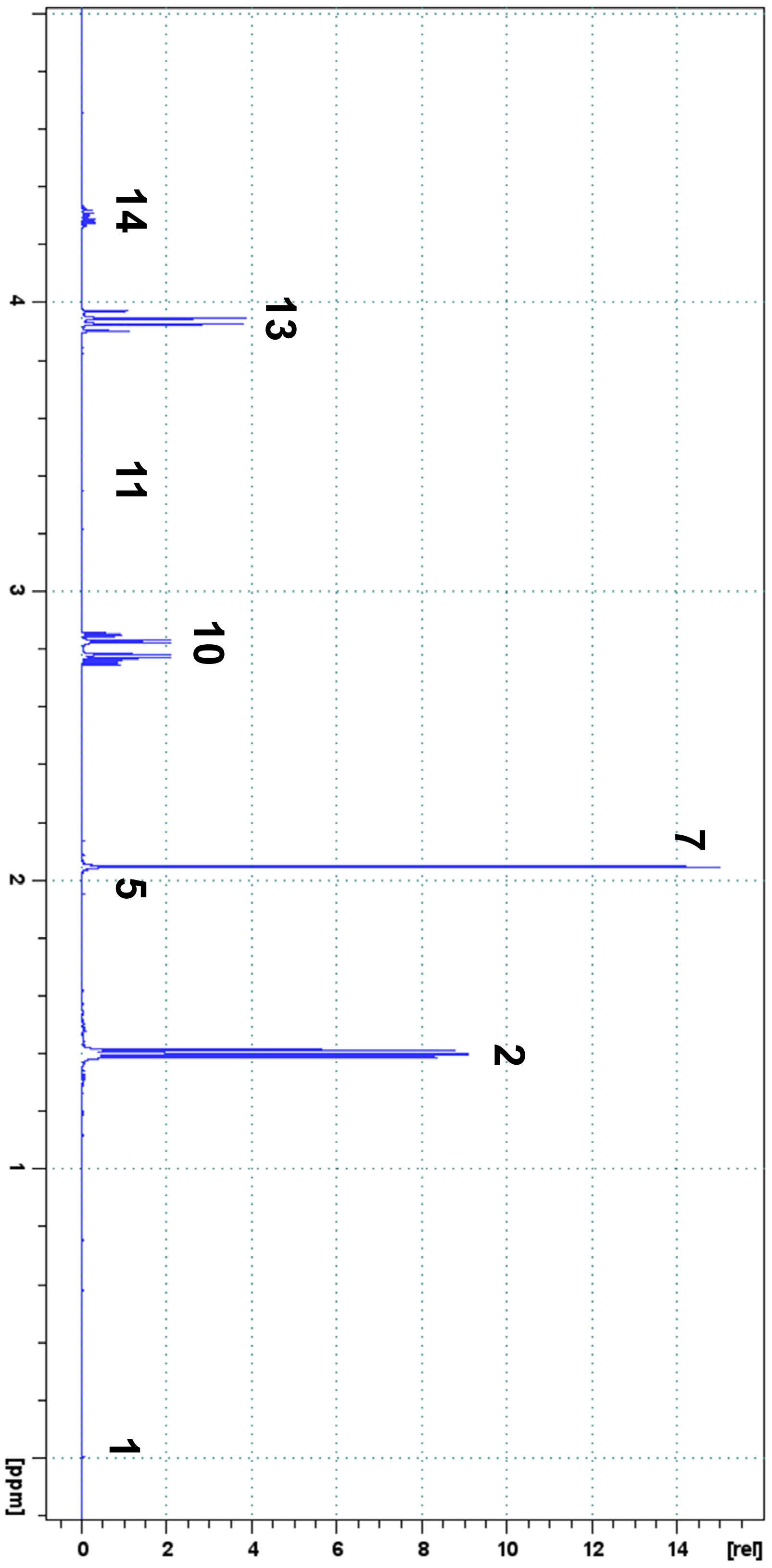

Supplementary Data S1 Figure S2 1D ^{1}H-NMR spectrum of the products of the isomerization reaction of deuterated Ac-NGAA-NH_2 (Ac-αDGAA-NH_2 and Ac-βDGAA-NH_2) measured at 700 MHz, 37 °C, in 50 mM Na_2HPO_4 buffer in D_2O, with the pH adjusted to 7.0, taking into account the effect of proton-deuterium exchange[3] (corresponds to pH 7.4 in H_2O).

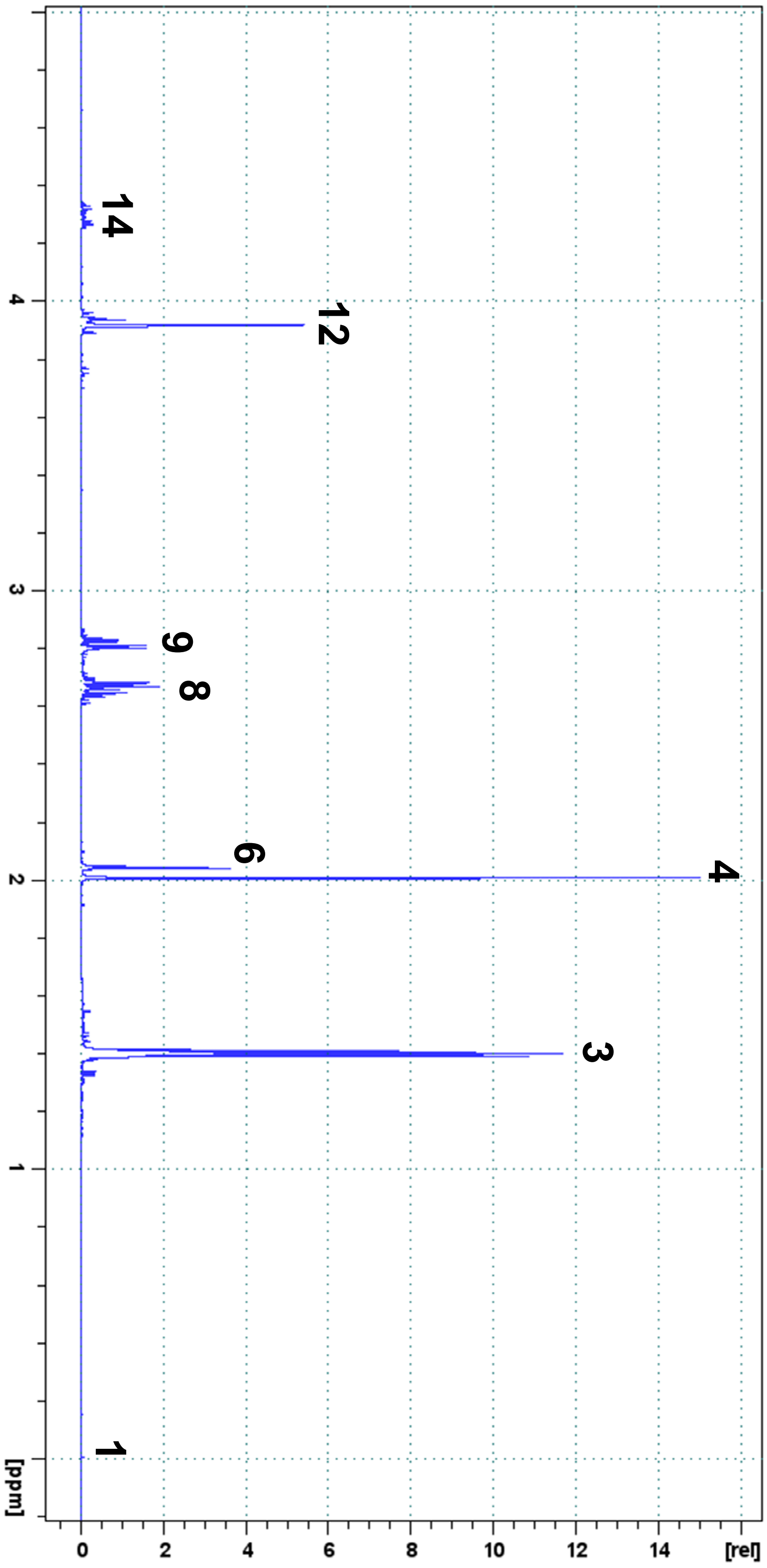

Supplementary Data S1 Figure S3 Enlargements of 1D ^{1}H-NMR spectra of deuterated Ac-NGAA-NH_2, and the products of its isomerization reaction (Ac-*α*DGAA-NH_2 and Ac-*β*DGAA-NH_2), measured at 700 MHz, 37 °C, in 50 mM Na_2HPO_4 buffer in D_2O, with the pH adjusted to 7.0, taking into account the effect of proton-deuterium exchange[3] (corresponds to pH 7.4 in H_2O).

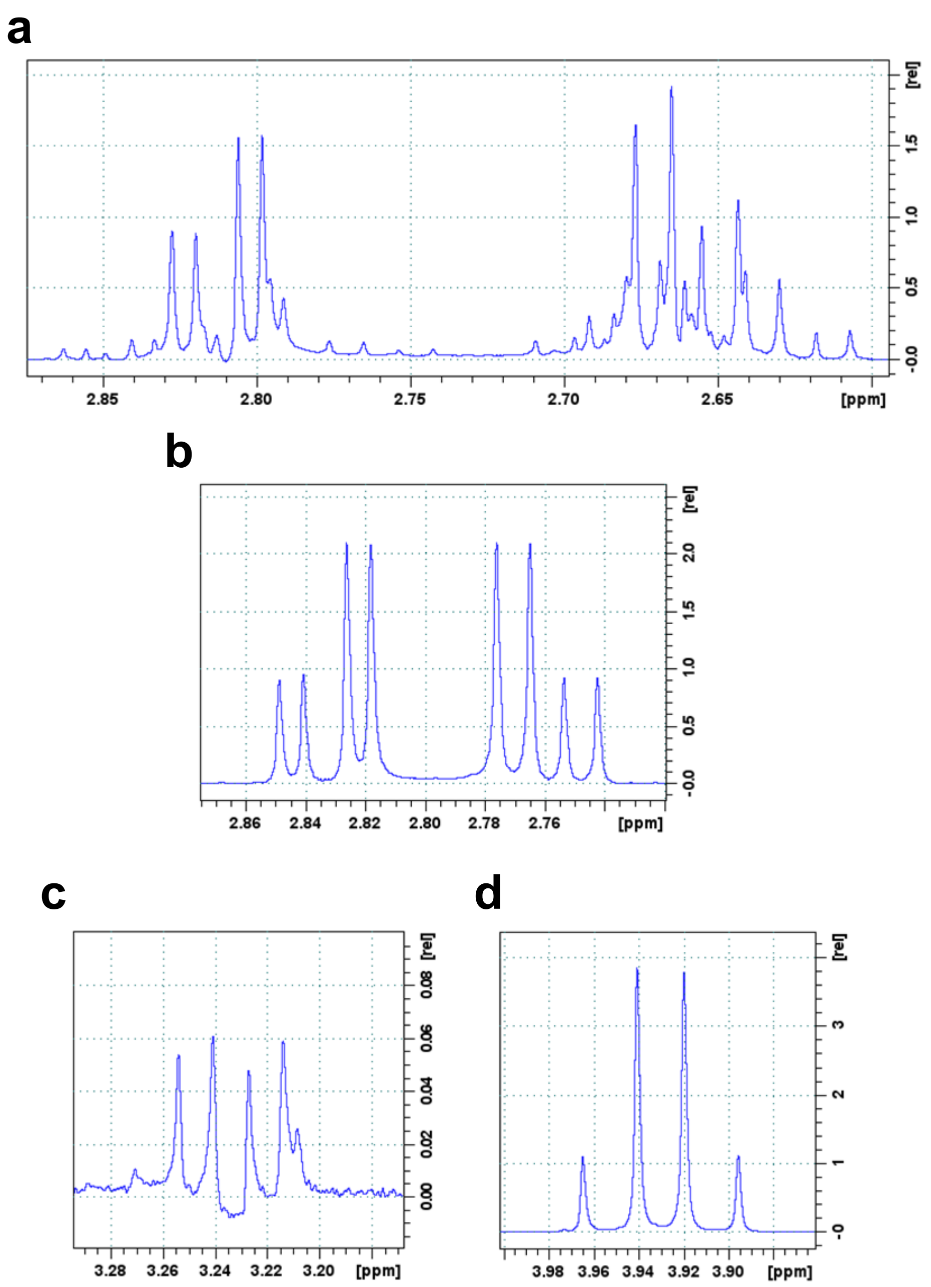

a Coupling pattern of β-ASP-HB and α-ASP-HB protons (8,9: **Supplementary Data S1 Note S2 and Supplementary Data S1 Figure S2**). **b** Coupling pattern of ASN-HB protons (10: **Supplementary Data S1 Note S2 and Supplementary Data S1 Figure S1**). **c** Coupling pattern of Suc-HB protons (11: **Supplementary Data S1 Note S2 and Supplementary Data S1 Figure S1**). **d** Coupling pattern of GLY-HA protons (13: **Supplementary Data S1 Note S2 and Supplementary Data S1 Figure S1**).

Supplementary Data S1 Note S3: Signal assignment and coupling constants of deuterated Ac-NGEA-NH_2 peptide and the products of its isomerization reaction (Ac-*α*DGEA-NH_2 and Ac-*β*DGEA-NH_2) by 1D ^{1}H-NMR spectra at 700 MHz, 37 °C, in 50 mM Na_2HPO_4 buffer in D_2O, with the pH adjusted to 7.0, taking into account the effect of proton-deuterium exchange[3] (corresponds to pH 7.4 in H_2O).

1: δ(DSS) = 0.0000 ppm (s)

2: δ(ALA-HB) = 1.3984 ppm (d), ^{3}J(ALA-HB:HA) = 7.34 Hz, of molecules containing β-Asp.
3: δ(ALA-HB) = 1.4035 ppm (d), ^{3}J(ALA-HB:HA) = 7.34 Hz, of molecules containing Asn.
4: δ(ALA-HB) = 1.4063 ppm (d), ^{3}J(ALA-HB:HA) = 7.34 Hz, of molecules containing α-Asp.

5;9: Composite multiplets: δ = 1.9455 ppm (m) and 2.0786 ppm (m) composed of GLU-HB2 and GLU-HB3 signals, of molecules containing either Asn, α-Asp or β-Asp.

6: δ(β-Asp-acetyl-H) = 1.9990 ppm (s)
7: δ(α-Asp-acetyl-H) = 2.0348 ppm (s)
8: δ(Asn-acetyl-H) = 2.0451 ppm (s)

10: δ(GLU-HG) = 2.2546 ppm (m), molecules containing either Asn, α-Asp or β-Asp.

11: δ(α-ASP-HB) = 2.83814 (dd*), 2.5994 ppm (dd), ^{2}J(α-ASP-HB2:HB3) = 16.05 Hz, ^{3}J(α-ASP-HB:HA) = 8.25 Hz *(only two of the expected peaks are visible, due to overlap with β-ASP-HB signals)
12: δ(β-ASP-HB) = 2.8337 ppm (dd), 2.6709 ppm (dd), ^{2}J(β-ASP-HB2:HB3) = 15.22 Hz, ^{3}J(β-ASP-HB:HA) = 4.87 Hz, 8.85 Hz
13: δ(ASN-HB) = 2.8401 ppm (dd), 2.7661 ppm (dd), ^{2}J(ASN-HB2:HB3) = 15.82 Hz, ^{3}J(ASN-HB:HA) = 5.55 Hz, 7.87 Hz

14: δ(Suc-HB) = 3.2423 ppm (m)

15: δ(GLY-HA) = 3.9782 ppm (d), 3.9133 ppm (d), ^{2}J(GLY-HA2:HA3) = 16.95 Hz, signals corresponding to molecules containing Asn.

16: Composite multiplet: δ = 3.9411 ppm (m), composed of GLY-HA signals of molecules containing either α-Asp or β-Asp.

17: Composite multiplet: δ = 4.2764 ppm (m), composed of ALA-HA (q) and GLU-HA (dd) signals of molecules containing either Asn, α-Asp or β-Asp.

In the NH region no signals are expected due to proton-deuterium exchange. However, low intensity signals are observed due to incomplete deuteration.

ASN-HA signal is expected to be within the water suppression region, and accordingly cannot be seen in the spectrum.

Supplementary Data S1 Figure S4 1D ^{1}H-NMR spectrum of deuterated Ac-NGEA-NH_2 measured at 700 MHz, 37 °C, in 50 mM Na_2HPO_4 buffer in D_2O, with the pH adjusted to 7.0, taking into account the effect of proton-deuterium exchange[3] (corresponds to pH 7.4 in H_2O).

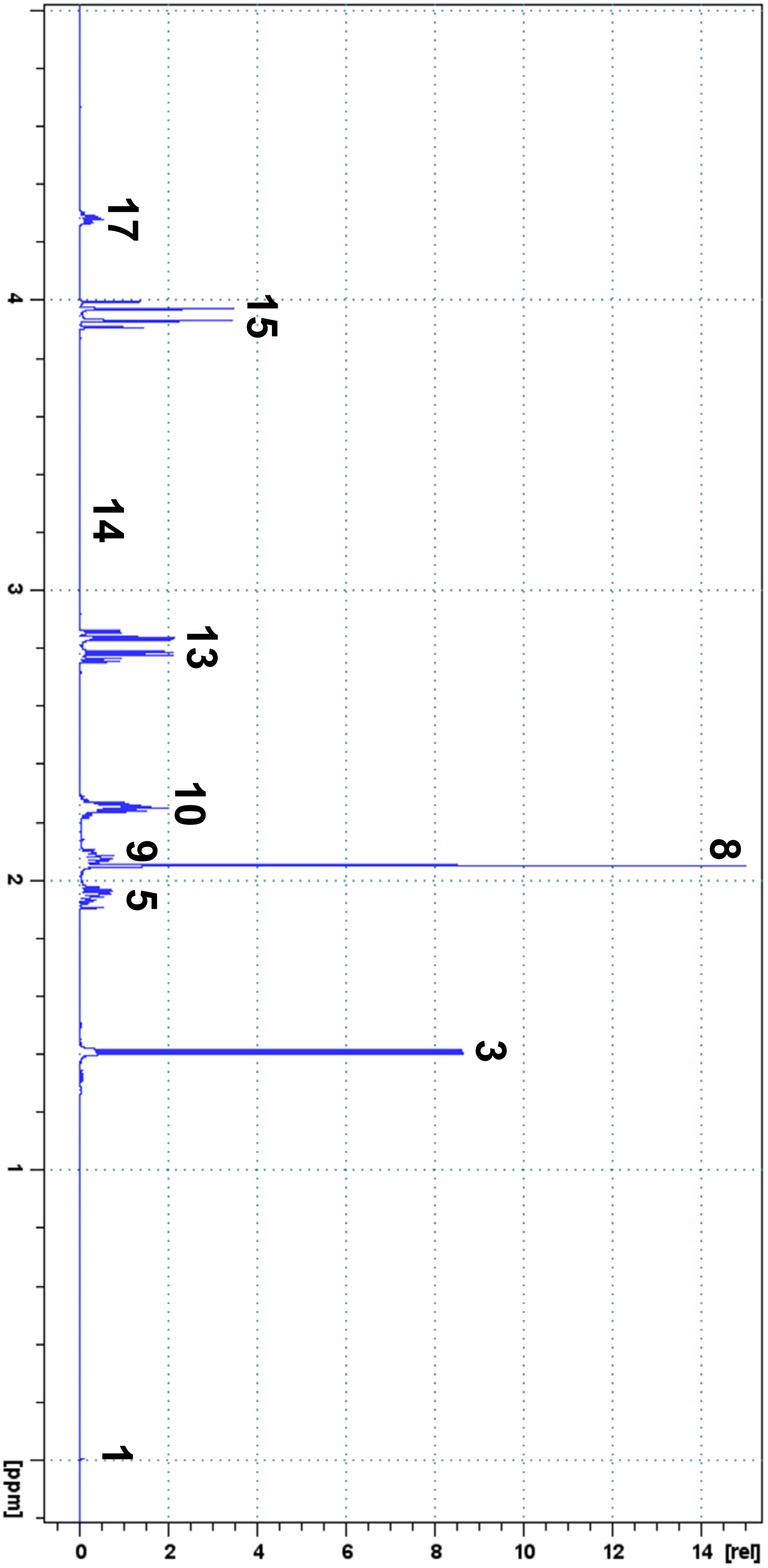

Supplementary Data S1 Figure S5 1D ^{1}H-NMR spectrum of deuterated Ac-NGEA-NH_2, and the products of its isomerization reaction (Ac-*α*DGEA-NH_2 and Ac-*β*DGEA-NH_2) measured at 700 MHz, 37 °C, in 50 mM Na_2HPO_4 buffer in D_2O, with the pH adjusted to 7.0, taking into account the effect of proton-deuterium exchange[3] (corresponds to pH 7.4 in H_2O).

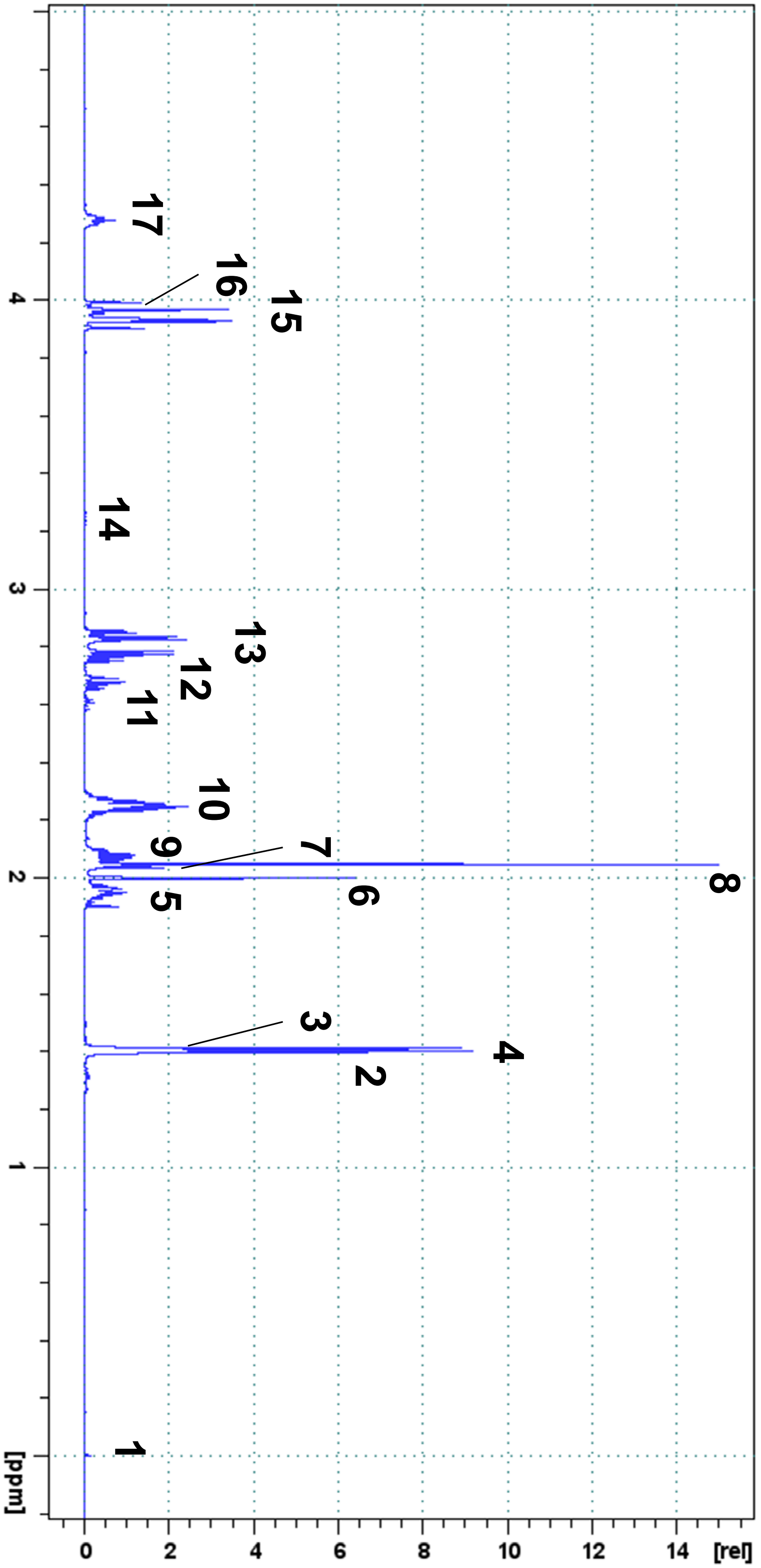

Supplementary Data S1 Figure S6 Enlargements of 1D ^{1}H-NMR spectra of deuterated Ac-NGEA-NH_2, and the products of its isomerization reaction (Ac-*α*DGEA-NH_2 and Ac-*β*DGEA-NH_2), measured at 700 MHz, 37 °C, in 50 mM Na_2HPO_4 buffer in D_2O, with the pH adjusted to 7.0, taking into account the effect of proton-deuterium exchange[3] (corresponds to pH 7.4 in H_2O).

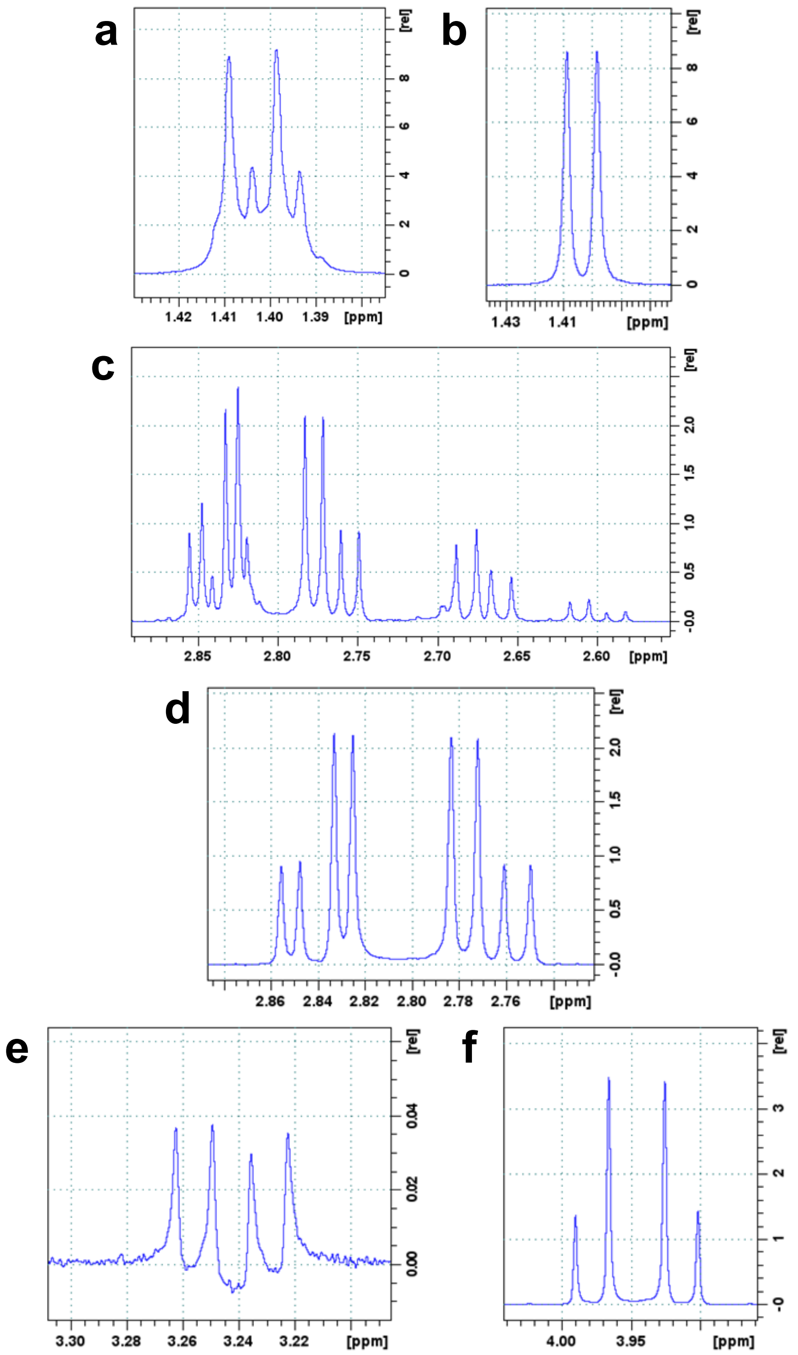

a Coupling pattern of ALA-HB protons in molecules containing Asn, α-Asp and β-Asp (2,3,4: **Supplementary Data S1 Note S3 and Supplementary Data S1 Figure S5**). **b** Coupling pattern of ALA-HB protons (3: **Supplementary Data S1 Note S3 and Supplementary Data S1 Figure S4**). **c** Coupling pattern of ASN-HB, α-ASP-HB and β-ASP-HB protons (11, 12, 13: **Supplementary Data S1 Note S3 and Supplementary Data S1 Figure S5**). **d** Coupling pattern of ASN-HB protons (13: **Supplementary Data S1 Note S3 and Supplementary Data S1 Figure S4**). **e** Coupling pattern of Suc-HB protons (14: **Supplementary Data S1 Note S3 and Supplementary Data S1 Figure S4**). **f** Coupling pattern of GLY-HA protons (15: **Supplementary Data S1 Note S3 and Supplementary Data S1 Figure S4**).

Supplementary Data S1 References

1. Chemical shift references. https://bmrb.io/ref_info/stats.php?restype=aa&set=full

2. Chemical shift reference for acetyl group. https://www.ucl.ac.uk/nmr/sites/nmr/files/L2_3_web.pdf

3. Glasoe, P. K. & Long, F. A. USE OF GLASS ELECTRODES TO MEASURE ACIDITIES IN DEUTERIUM OXIDE[1,2]. *J. Phys. Chem.* **64**, 188–190 (1960).
