# Supplementary material for: Impact of Structural Flexibility on Tunneling Contribution to the Reaction Mechanism of Spontaneous Succinimide Formation in Asn-Gly-Containing Peptides
Source: Biomolecules. 2026 Sep 1;16(9):1266. doi: 10.3390/biom16091266 (PMC13604283; doi:10.3390/biom16091266)
Supplement: Supplementary file 1 [file biomolecules-16-01266-s001.zip › Supplementary Data S2.pdf]

Supplementary Data S2

Input parameters, coordinates and absolute energies of the structures used in IRC path calculations, structure optimizations and analysis.

Impact of Structural Flexibility on Tunneling Contribution to the Reaction Mechanism of Spontaneous Succinimide Formation in Asn-Gly-Containing Peptides

Fruzsina Pilhál [1,2], Bianka Szalainé Ágoston [1,3], Imre Jákli [1,4], Ernő Keszei [5] and András Perczel [1,4,*]

1 F. Pilhál, Dr. B. Szalainé Ágoston, Dr. I. Jákli, Prof. A. Perczel
Laboratory of Structural Chemistry and Biology, Institute of Chemistry
ELTE Eötvös Loránd University
Pázmány Péter sétány. 1/A, 1117 Budapest, Hungary
* E-mail: A.P. perczel.andras@ttk.elte.hu

2 F. Pilhál
Hevesy György PhD School of Chemistry
ELTE Eötvös Loránd University
Pázmány Péter sétány. 1/A, 1117 Budapest, Hungary

3 B. Szalainé Ágoston
Present address: Faculty of Information Technology and Bionics
Pázmány Péter Catholic University
Práter utca 50/a, 1083 Budapest, Hungary

4 Dr. I. Jákli, Prof. A. Perczel
HUN-REN–ELTE Protein Modelling Research Group, Institute of Chemistry
ELTE Eötvös Loránd University
Pázmány Péter sétány. 1/A, 1117 Budapest, Hungary

5 Prof. E. Keszei
Department of Physical Chemistry and Chemical Kinetics Laboratory, Institute of Chemistry
ELTE Eötvös Loránd University
Pázmány Péter sétány. 1/A, 1117 Budapest, Hungary

Supplementary Data S2 Note S1: Input parameters for IRC path calculations.

For IRC path calculations, the following parameters were set.

```
#p irc=(calcall,maxpoints=100,maxcycle=300,readiso) b3lyp/6-31+g(d,p)
```

```
#p irc=(calcall,maxpoints=30,maxcycle=300,readiso) b3lyp/6-31+g(d,p)
```

Supplementary Data S2 Table S1 Cartesian coordinates and input parameters of open form of the deuterated system in vacuum.

#p opt freq b3lyp/6-311++g(d,p)

0 1

C	4.31229000	0.45100900	0.62492200
C	3.04142400	-0.24248300	0.16085600
O	3.04051600	-1.42561000	-0.17942100
N	1.90898200	0.52500100	0.17354100
C	0.64242100	0.11660000	-0.43304900
C	-0.25378000	1.37851100	-0.46131000
O	0.23449300	2.45209500	-0.81682100
C	0.04558900	-1.15417500	0.21735900
C	-1.20098700	-1.64057100	-0.51401600
N	-2.29536700	-2.01524400	0.31140800
O	-1.30029600	-1.67797900	-1.72431900
N	-1.56926500	1.20369100	-0.19138700
C	-2.52793000	2.27545900	-0.42244000
O	-2.53659700	0.08627300	2.23282400
H	4.15028400	1.47338400	0.97663200
H	5.02280100	0.46695400	-0.20702400
H	4.76073600	-0.13962600	1.42818800
H(Iso=2)	2.01800600	1.53167400	0.22908700
H	0.80345500	-0.12683400	-1.49304600
H	0.81391500	-1.93132600	0.13227100
H	-0.15128900	-0.99214000	1.28134100
H	-2.31871000	3.16263200	0.18590000
H	-3.52248900	1.89803800	-0.17855200
H	-2.50165100	2.56729400	-1.47498300
H(Iso=2)	-2.64975400	-0.77824000	1.79453000
H(Iso=2)	-2.09811200	-0.05800300	3.07935200
H(Iso=2)	-1.80764100	0.58985300	0.58033700
H(Iso=2)	-2.05173000	-2.70598700	1.01877700
H(Iso=2)	-3.06679100	-2.34633300	-0.26404800

Supplementary Data S2 Table S2 Cartesian coordinates and energy value of iteration step 43 of the open form of the deuterated system in vacuum at DFT B3LYP/6-311++G(d,p) level of theory.

Along the optimization of the open form, an oscillatory cycle developed. Therefore, in order to reach the energy minimum, the optimization process was restarted from iteration step 43, which had not yet been affected by the oscillation. Below, we present the Cartesian coordinates and energy value of the structure obtained at iteration step 43.

Total Energy = -741.2344557 Hartree

0 1

C	4.26264400	-0.23290700	0.61979400
C	2.90799300	-0.51834000	-0.00122700
O	2.72400600	-1.47752600	-0.74451900
N	1.91278900	0.34917300	0.32334900
C	0.59458100	0.28745200	-0.28057500
C	-0.07790700	1.64858600	0.01082900
O	0.43290500	2.44477600	0.79657200
C	-0.21074900	-0.91537400	0.27196000
C	-1.29088000	-1.42901600	-0.66661300
N	-1.80483700	-2.67311900	-0.34929200
O	-1.74319600	-0.79220900	-1.60484200
N	-1.23124400	1.87770200	-0.63938300
C	-2.02127600	3.07694700	-0.40393800
O	-3.28718800	-1.19208700	1.94204600
H	4.24986600	0.60068800	1.32433900
H	4.97176600	-0.01131000	-0.18138800
H	4.61378600	-1.13230800	1.12899500
H(Iso=2)	2.08040600	1.17847900	0.88186900
H	0.70049700	0.15828900	-1.36166000
H	0.49529700	-1.72566800	0.46604400
H	-0.69098800	-0.66145400	1.22396400
H	-1.39786100	3.96674600	-0.50611900
H	-2.45687500	3.08061400	0.59999300
H	-2.82319400	3.11425000	-1.14067400
H(Iso=2)	-3.06971400	-1.83984000	1.25353900
H(Iso=2)	-3.62643900	-1.69784100	2.68648400

H(Iso=2)	-1.59296000	1.13670500	-1.22836600
H(Iso=2)	-1.21749300	-3.31313300	0.16704300
H(Iso=2)	-2.37170200	-3.09822600	-1.07221500

Supplementary Data S2 Table S3 Cartesian coordinates and input parameters of iteration step 43 of the open form of the deuterated system in vacuum.

Along the optimization of the open form, an oscillatory cycle developed. Therefore, in order to reach the energy minimum, the optimization process was restarted from iteration step 43, which had not yet been affected by the oscillation. Below, we present the Cartesian coordinates and input parameters of iteration step 43 as a new starting point for the optimization.

```
#p opt freq b3lyp/6-311++g(d,p)
0 1
```

C	4.26264400	-0.23290700	0.61979400
C	2.90799300	-0.51834000	-0.00122700
O	2.72400600	-1.47752600	-0.74451900
N	1.91278900	0.34917300	0.32334900
C	0.59458100	0.28745200	-0.28057500
C	-0.07790700	1.64858600	0.01082900
O	0.43290500	2.44477600	0.79657200
C	-0.21074900	-0.91537400	0.27196000
C	-1.29088000	-1.42901600	-0.66661300
N	-1.80483700	-2.67311900	-0.34929200
O	-1.74319600	-0.79220900	-1.60484200
N	-1.23124400	1.87770200	-0.63938300
C	-2.02127600	3.07694700	-0.40393800
O	-3.28718800	-1.19208700	1.94204600
H	4.24986600	0.60068800	1.32433900
H	4.97176600	-0.01131000	-0.18138800
H	4.61378600	-1.13230800	1.12899500
H(Iso=2)	2.08040600	1.17847900	0.88186900
H	0.70049700	0.15828900	-1.36166000
H	0.49529700	-1.72566800	0.46604400
H	-0.69098800	-0.66145400	1.22396400
H	-1.39786100	3.96674600	-0.50611900
H	-2.45687500	3.08061400	0.59999300
H	-2.82319400	3.11425000	-1.14067400
H(Iso=2)	-3.06971400	-1.83984000	1.25353900
H(Iso=2)	-3.62643900	-1.69784100	2.68648400
H(Iso=2)	-1.59296000	1.13670500	-1.22836600

H(Iso=2)	-1.21749300	-3.31313300	0.16704300
H(Iso=2)	-2.37170200	-3.09822600	-1.07221500

Supplementary Data S2 Table S4 Cartesian coordinates and energy values of DFT B3LYP/6-311++G(d,p) optimized open form of the deuterated system in vacuum.

Sum of electronic and thermal Enthalpies = -741.000365 Hartree

Sum of electronic and thermal Free Energies = -741.072309 Hartree

Total Energy = -741.2381999 Hartree

0 1

C	-0.93197100	-3.88728700	0.14562900
C	-0.81356600	-2.41592300	-0.18593300
O	-1.71375300	-1.80836700	-0.76908000
N	0.32741900	-1.79625100	0.20519000
C	0.58309700	-0.41558800	-0.14608200
C	2.06476500	-0.10990700	0.14896600
O	2.81978300	-0.98460000	0.55884800
C	-0.39092700	0.57098600	0.57299000
C	-0.89778200	1.58024100	-0.43929300
N	-2.08572200	1.24399300	-1.03473900
O	-0.26673900	2.59083800	-0.72177300
N	2.44134400	1.16099700	-0.10234200
C	3.80452700	1.61773000	0.13460400
O	-3.93892500	2.00904600	1.36703300
H	-0.05699500	-4.28467700	0.66216200
H	-1.08670000	-4.44251800	-0.78181700
H	-1.81629900	-4.03627200	0.76859400
H(Iso=2)	1.11713200	-2.30059400	0.59152900
H	0.44388500	-0.31040900	-1.22872600
H	-1.21677100	0.00754600	1.00149600
H	0.11489900	1.10586900	1.37703200
H	4.51066900	0.86136000	-0.20823200
H	3.98999200	1.80322300	1.19701200
H	3.96587200	2.54136600	-0.42090400
H(Iso=2)	-3.47541000	1.88393600	0.52692100
H(Iso=2)	-4.07468200	2.95832000	1.44110700
H(Iso=2)	1.73423700	1.84182400	-0.36533800
H(Iso=2)	-2.32850200	0.25431600	-1.02754400
H(Iso=2)	-2.31440100	1.75868300	-1.87556300

Supplementary Data S2 Table S5 Cartesian coordinates and input parameters of transition state of the deuterated system in vacuum.

#p opt=(calcall,ts,noeigentest) freq b3lyp/6-311++g(d,p)

0 1

C	4.29564800	0.42459800	0.63372100
C	3.02223900	-0.25684700	0.16419000
O	3.01118900	-1.44279700	-0.17499900
N	1.89749600	0.51552900	0.16484000
C	0.64958400	0.10541700	-0.45915600
C	-0.32199100	1.30595100	-0.42710800
O	0.15061500	2.42602500	-0.70150400
C	0.04259100	-1.15973200	0.17418700
C	-1.22232400	-1.50994000	-0.55113900
N	-2.42411400	-1.83801000	0.38204300
O	-1.37817500	-1.78355600	-1.70787400
N	-1.59719700	0.98587500	-0.15486200
C	-2.55866600	2.05278700	-0.46023800
O	-2.45244800	0.08763800	2.11442000
H	4.13574000	1.44397100	0.99469900
H	5.00487200	0.44761700	-0.19937200
H	4.74426200	-0.17529800	1.42997200
H(Iso=2)	1.98394800	1.52591400	0.22748000
H	0.82967200	-0.12165700	-1.52010300
H	0.75321700	-1.98497100	0.04306800
H	-0.13667800	-1.00939200	1.24059200
H	-2.34880600	2.97187400	0.10337700
H	-3.56764300	1.71303700	-0.20455600
H	-2.53532200	2.31736300	-1.52477700
H(Iso=2)	-2.46202800	-1.18156100	1.24079200
H(Iso=2)	-1.99340300	0.30513800	2.93408100
H(Iso=2)	-1.99914800	0.57655400	1.30494300
H(Iso=2)	-2.36270100	-2.81620000	0.67792000
H(Iso=2)	-3.27119400	-1.72802200	-0.17933900

Supplementary Data S2 Table S6 Cartesian coordinates and energy values of DFT B3LYP/6-311++G(d,p) transition state of the deuterated system in vacuum.

Sum of electronic and thermal Enthalpies = -740.924692 Hartree

Sum of electronic and thermal Free Energies = -740.987405 Hartree

Total Energy = -741.1580152 Hartree

0 1

C	4.28581200	0.41789600	0.64310200
C	3.01357100	-0.26009000	0.16934300
O	3.00154200	-1.43786700	-0.17377400
N	1.89212500	0.51449200	0.17660500
C	0.64005500	0.11328600	-0.44400100
C	-0.31917100	1.32622400	-0.41482400
O	0.17297200	2.43182300	-0.68669400
C	0.03628900	-1.15136400	0.19103300
C	-1.19797000	-1.54460700	-0.55676000
N	-2.40546500	-1.89836600	0.36477400
O	-1.33809000	-1.79711300	-1.71016900
N	-1.59742900	1.02854600	-0.15246200
C	-2.53367200	2.12076000	-0.44419800
O	-2.51907100	0.05782600	2.04813700
H	4.13336600	1.44765600	0.97039500
H	5.00944500	0.40530800	-0.17509600
H	4.70970500	-0.16290900	1.46452700
H(Iso=2)	1.98140700	1.52253800	0.24478200
H	0.81523600	-0.11344500	-1.50367900
H	0.76540400	-1.96304100	0.08592300
H	-0.17066300	-0.99376900	1.24936500
H	-2.29446500	3.03167100	0.11750300
H	-3.54647700	1.80644800	-0.17960200
H	-2.51549300	2.38720000	-1.50658300
H(Iso=2)	-2.48557000	-1.21547100	1.20074000
H(Iso=2)	-2.12199000	0.31440100	2.88497300
H(Iso=2)	-2.04705400	0.55988400	1.25307400
H(Iso=2)	-2.29976900	-2.85760900	0.70138000
H(Iso=2)	-3.24599700	-1.85154400	-0.21229600

Supplementary Data S2 **Table S7** Cartesian coordinates and input parameters of succinimide of the deuterated system in vacuum.

#p opt freq b3lyp/6-311++g(d,p)

0 1

C	4.37683900	0.46389800	0.69239700
C	3.11794700	-0.22537700	0.19991400
O	3.11562400	-1.39965200	-0.16597800
N	1.98256600	0.53575800	0.20054700
C	0.78605300	0.09229900	-0.48255000
C	-0.24391800	1.22202200	-0.46603700
O	0.08201200	2.40586900	-0.60256500
C	0.07602900	-1.13650600	0.09210900
C	-1.28035700	-1.07755500	-0.61124500
N	-2.46237400	-1.68319600	0.41181300
O	-1.48020400	-1.49483400	-1.74610900
N	-1.49552200	0.71383800	-0.29313700
C	-2.60236700	1.61229600	-0.65493700
O	-2.36117900	0.23790100	2.32809800
H	4.19885500	1.47129000	1.07843200
H	5.08840700	0.52051100	-0.13716800
H	4.83320600	-0.14774800	1.47494900
H(Iso=2)	2.05774500	1.54413000	0.28541000
H	1.01445800	-0.11862800	-1.53780800
H	0.59606200	-2.06213600	-0.15914500
H	-0.01408300	-1.04426100	1.17901600
H	-2.44737900	2.58462100	-0.17651900
H	-3.55387300	1.19925900	-0.30835600
H	-2.65514900	1.77326900	-1.73794900
H(Iso=2)	-2.51019900	-1.21382800	1.33384700
H(Iso=2)	-1.93179300	0.50755500	3.14829300
H(Iso=2)	-1.88291300	0.66350100	1.57790500
H(Iso=2)	-2.28615600	-2.68337100	0.50878900
H(Iso=2)	-3.33750300	-1.56851600	-0.09933500

Supplementary Data S2 Table S8 Cartesian coordinates and energy values of DFT B3LYP/6-311++G(d,p) optimized succinimide of the deuterated system in vacuum.

Sum of electronic and thermal Enthalpies = -741.007298 Hartree

Sum of electronic and thermal Free Energies = -741.080685 Hartree

Total Energy = -741.2440033 Hartree

0 1

C	3.60127800	-1.05307100	0.18536200
C	2.23096400	-1.37604200	-0.37622600
O	2.07786100	-2.20006100	-1.26770800
N	1.18784400	-0.69150000	0.18581600
C	-0.15256200	-0.89276500	-0.29659300
C	-0.88597100	0.40717100	-0.64104800
O	-0.43933500	1.39536000	-1.18935900
C	-1.13069300	-1.62488300	0.64432800
C	-2.45440100	-0.90324300	0.47561000
N	1.67596900	1.61117900	2.10294700
O	-3.53932800	-1.22489200	0.89280500
N	-2.20577300	0.26993300	-0.25520500
C	-3.22661900	1.26963200	-0.54386600
O	1.69392800	3.13688300	-0.48709500
H	3.57336100	-0.31309000	0.98550900
H	4.23043100	-0.68379300	-0.62723700
H	4.05248300	-1.97564700	0.55620000
H(Iso=2)	1.36355700	0.02303400	0.90219600
H	-0.05645100	-1.45307900	-1.23358500
H	-1.24395200	-2.68700600	0.42723900
H	-0.81928100	-1.52985000	1.68840300
H	-3.25116600	1.47390500	-1.61468300
H	-3.00818800	2.19828100	-0.01373800
H	-4.18328500	0.86933400	-0.21371300
H(Iso=2)	1.86626800	2.29560100	1.36716000
H(Iso=2)	2.20244900	3.52547400	-1.20375600
H(Iso=2)	1.03903500	2.55207900	-0.90483000
H(Iso=2)	2.48488700	1.58319000	2.71713900
H(Iso=2)	0.90659500	1.97516700	2.65824800
